# New MicroRNAs in *Drosophila*—Birth, Death and Cycles of Adaptive Evolution

**DOI:** 10.1371/journal.pgen.1004096

**Published:** 2014-01-23

**Authors:** Yang Lyu, Yang Shen, Heng Li, Yuxin Chen, Li Guo, Yixin Zhao, Eric Hungate, Suhua Shi, Chung-I Wu, Tian Tang

**Affiliations:** 1 State Key Laboratory of Biocontrol, Key Laboratory of Gene Engineering of the Ministry of Education, School of Life Sciences, Sun Yat-sen University, Guangzhou, Guangdong, China; 2 Guangdong Key Laboratory of Plant Resources, School of Life Sciences, Sun Yat-sen University, Guangzhou, Guangdong, China; 3 Key Laboratory of Biodiversity Dynamics and Conservation of Guangdong Higher Education Institutes, Sun Yat-sen University, Guangzhou, Guangdong, China; 4 Department of Ecology and Evolution, University of Chicago, Chicago, Illinois, United States of America; 5 Beijing Institute of Genomics, Chinese Academy of Sciences, Beijing, China; Fred Hutchinson Cancer Research Center, United States of America

## Abstract

The origin and evolution of new microRNAs (miRNAs) is important because they can impact the transcriptome broadly. As miRNAs can potentially emerge constantly and rapidly, their rates of birth and evolution have been extensively debated. However, most new miRNAs identified appear not to be biologically significant. After an extensive search, we identified 12 new miRNAs that emerged *de novo* in *Drosophila melanogaster* in the last 4 million years (Myrs) and have been evolving adaptively. Unexpectedly, even though they are adaptively evolving at birth, more than 94% of such new miRNAs disappear over time. They provide selective advantages, but only for a transient evolutionary period. After 30 Myrs, all surviving miRNAs make the transition from the adaptive phase of rapid evolution to the conservative phase of slow evolution, apparently becoming integrated into the transcriptional network. During this transition, the expression shifts from being tissue-specific, predominantly in testes and larval brain/gonads/imaginal discs, to a broader distribution in many other tissues. Interestingly, a measurable fraction (20–30%) of these conservatively evolving miRNAs experience “evolutionary rejuvenation” and begin to evolve rapidly again. These rejuvenated miRNAs then start another cycle of adaptive – conservative evolution. In conclusion, the selective advantages driving evolution of miRNAs are themselves evolving, and sometimes changing direction, which highlights the regulatory roles of miRNAs.

## Author Summary

During Metazoan evolution, the architecture of the genome changed dramatically in size, gene number and regulatory elements. Genomic architecture is often assumed to be correlated with morphological complexity. However, it is still not known whether the gene repertoire, both for protein coding and non-coding genes, is continually increasing. In the last decade, a large family of small non-coding RNAs, or microRNAs (miRNAs), has been shown to play an important role in diverse developmental processes. The genes controlled by miRNAs often evolve rapidly, potentially contributing to functional novelty, diversity and speciation. Here we estimated the birth and death rate of new adaptive miRNAs in *Drosophila melanogaster*. We found most new adaptive miRNAs disappear over long periods of time; hence, the miRNA repertoire stays close to that of a steady state. This steady state is commensurate with the morphological constancy of the genus of *Drosophila*.

## Introduction

MicroRNAs (miRNAs) are a class of small, endogenous RNAs that regulate gene expression post-transcriptionally [1], [2]. Each miRNA gene is first transcribed as a stem-loop (hairpin) RNA structure, 70–90 nt in length in animals, and then processed in several steps into the ∼22-nt mature product, referred to as miR [3]. In animals, miR binds to the 3′ untranslated region (UTR) of target mRNAs through perfect base-pairing of the seed region (position 2–8 of a miR), inducing translation repression or mRNA degradation [4]. As the seed is only 7 nt long, each miRNA may potentially regulate hundreds of transcripts while each transcript may in turn be regulated by more than one miRNA [5].

The emergence of new miRNAs is of special interest in evolutionary biology for two reasons. First, they buffer gene expression noises and thus have been hypothesized to be a key player in canalization [6], [7]. As proposed by C. H. Waddington [8], [9], canalization contributes to developmental stability and, in a recent interpretation, it may also contribute to evolvability via hidden genetic variations [10], [11]. Second, due to their small size, miR-producing hairpins can form readily and *de novo* emergence of miRNAs from non-miRNA transcripts is a frequent phenomenon [12], [13]. There are hundreds of thousands of potential miRNA structures in each *Drosophila* genome [12] and millions in a mammalian genome [14]. Given such a propensity for new miRNAs to emerge, the birth, death and adaptation of new miRNAs are a significant part of understanding the evolution of transcriptional regulation [12]. In contrast, protein-coding genes require long open reading frames to yield functional peptides. Hence, local duplication or retrotransposition [15], rather than *de novo* origination, is the common mode for the formation of coding genes.

In *Drosophila*, the birth and death rates of miRNAs have been estimated to be about 12 and 11.7 genes per Myr, respectively, with a net gain of about 0.3 per Myr [12]. It is generally agreed that the net gain is low, ranging between 0.3 and 1 new gene per Myr [16], [17]. Despite this, the total repertoire of miRNAs should still be increasing dramatically over long periods. While the net gain (birth – death) is not in dispute, there is disagreement over the estimated birth and death rates of new miRNAs [12], [16], [17]. Because numerous putative miRNAs are found in the transcriptome, these lowly expressed, evolutionarily neutral, and short-lived miRNAs account for the bulk of the estimated births and deaths. The debate is about which ones should be counted as new miRNAs.

To resolve the issue, we propose to define new miRNAs in an evolutionary context by a set of stringent criteria, requiring a signature of initial adaptive evolution soon after their birth. Numerous small RNAs that emerge and vanish with the dynamics of neutral sequences are excluded from the evolutionary analysis. Given this definition, only a small fraction of miRNA-like sequences in any species would qualify as new miRNAs. We collected extensive small RNA-seq data available for four *Drosophila* species (*D. melanogaster*, *D. simulans*, *D. pseudoobscura* and *D. virilis*) [12], [16], [18]–[23] and three mosquitoes (*Aedes albopictus*, *Aedes aegypti* and *Culex quinquefasiatus*) [24], [25]. We further generated small RNA-seq data for sex organs and imaginal discs in *D. simulans* and *D. pseudoobscura.* The extensive dataset permits systematic identification of new miRNAs and in-depth analyses of their long-term fates.

Our first objective is to understand the origin and early evolution of new miRNAs in the species *D. melanogaster*. The second objective is to track the long-term evolutionary trajectory of new miRNAs, which may be in any of the following four modes after their initial adaptive evolution:

1. Evolving rapidly, driven by positive selection;
2. Transitioning between the initial adaptive phase and one of the two possible outcomes given below in 3) and 4). miRNAs in this phase may appear neutrally evolving;
3. Evolving conservatively and slowly after being assimilated into the transcriptional network;
4. Effectively dead after its structure degenerates and is no longer recognizable as an miRNA.

## Results

From the *D. melanogaster* miRNA repository (miRBase Release 19.0, Ref. [26]), 238 miRNA genes, including 204 canonical miRNAs and 34 mirtrons, were evaluated for their expression levels by examining small RNA sequencing data from different tissues and developmental stages (Ref. [12], [16], [18]–[21], [23], see **Table S1** and **Materials and Methods**). The phylogenetic distributions of the 238 miRNA genes in *Drosophila* (*D. melanogaster*, *D. simulans*, *D. pseudoobscura* and *D. virilis*), with mosquitoes (*Aedes albopictus*, *A. aegypti* and *Culex quinquefasiatus*) as the outgroup, were determined from the available small RNA libraries (Ref. [12], [16], [22], [24], [25], see **Table S1** and **Materials and Methods**). In addition, we sequenced five additional libraries from *D. simulans* and *D.pseudoobscura* to ensure that all *Drosophila* species in this survey included samples from testes and ovaries. Genes represented by more than 200 reads per million (RPM) in at least one library were designated “highly expressed” (**Table S2**). The rest were denoted as “lowly expressed” miRNAs.

**Figure 1:**
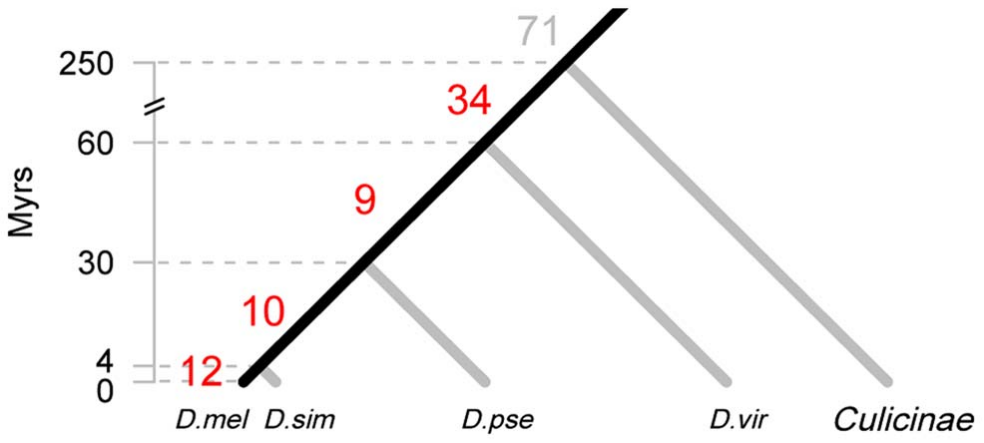
Origin of new miRNAs at different evolutionary periods. The divergence time of the phylogeny is based on the *Drosophila* 12 Genomes [27], Gaunt *et al.* [65] and Bolshakov *et al.* [66]. The number of highly expressed, canonical miRNAs that are inferred to originate in each time interval is given on the corresponding branch. Species abbreviations: *D. mel*, *D. melanogaster*; *D. sim*, *D. simulans*; *D. pse*, *D. pseudoobscura*; *D. vir*, *D. virilis*. The 12 miRNAs of the last 4 Myrs are miR-979 and miR-4966 from the miR-972s cluster, miR-983-2 and miR-984 from the miR-982s cluster, miR-954, miR-956, miR-971, miR-985, miR-990, miR-997, miR-1017 and miR-2279. The ten miRNAs emerged between 4 and 30 Myrs ago are miR-972, miR-978 and miR-2499 from the miR-972s cluster, miR-303, miR-982 and miR-983-1 from the miR-982s cluster, miR-992 and miR-2498 from the miR-310s cluster, miR-1001 and miR-2494.

The 204 canonical miRNAs and 34 mirtrons have very different patterns in age and expression level. **Table 1** shows the emergence time of each miRNA, which falls in the interval of 0–4, 4–30, 30–60, 60–250, and >250 Myrs before present as depicted in **Fig. 1**. More than half of the highly expressed, canonical miRNAs (71 out of 136) came from the oldest age group (>250 Myrs) but none of the mirtrons were from that group (**Table 1**), suggesting mirtrons contribute very little to miRNA repertoire over long periods of time. The result is consistent with previous findings that mirtrons have different evolutionary trajectories from canonical miRNAs [16]. The majority of the lowly expressed genes, both canonical miRNAs (60/68) and mirtrons (21/25), came from the young age group of 0–4 Myrs (**Table 1**), corroborating that lowly expressed miRNA genes are likely to be evolutionarily transient [12].

**Table 1:** Number of miRNAs in the genome of *D. melanogaster* in different age groups.

type	expression level	age (Myrs)				
		0–4	4–30	30–60	60–250	>250
canonical (n = 204)	>200 RPM	12	10	9	34	71
	≤200 RPM	60	2	3	3	0
mirtrons (n = 34)	>200 RPM	3	2	1	3	0
	≤200 RPM	21	3	1	0	0

In this study, we will focus on the 136 highly expressed canonical miRNAs because, with respect to long-term evolution, they are the most significant class among the four categories of **Table 1**.

### I. Birth of new miRNAs

Starting with the youngest genes, we first analyzed the 22 new miRNA genes that emerged in the last 30 Myrs, since *D. melanogaster* diverged from *D. pseudoobscura* (**Fig. 1**). Among them, 21 originated *de novo*; only miR-983-2 in *D. melanogaster* (dme-miR-983-2) was duplicated from another miRNA (dme-miR-983-1). More than half of the 22 new miRNAs are found in clusters – five in the miR-972 cluster (abridged as miR-972s), two in miR-310s and five in miR-982s. Members in a cluster have significantly higher expression levels than the orphan miRNAs (Mann-Whitney U test, *p*<0.05). The miR-982 cluster consists only of members emerging in the last 30 Myrs, whereas both miR-310s and miR-972s are mixtures of old and new miRNAs (**Table S3**). Thus, the former is most informative about the birth and early evolution of new miRNAs.

**Figure 2:**
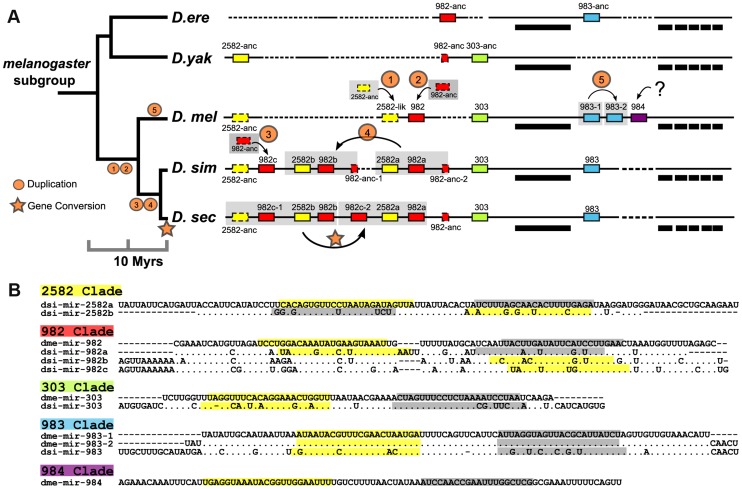
The evolution of the structure of the miR-982s cluster. (**A**) This cluster comprises members of five miRNA families (miR-982, miR-2582, miR-303, miR-983 and miR-984), indicated by red, yellow, green, blue and purple boxes, respectively. Boxes outlined with solid and dashed lines represent miRNA homologs with and without expression, respectively. Bold bars indicate *CG3626* exons 1–6 located on the opposite strand of miR-982s. Alignment gaps are illustrated with dashed lines. The genomic region is not drawn to scale. Species abbreviations: *D. ere*, *D. erecta*; *D. yak*, *D. yakuba*; *D. mel*, *D. melanogaster*; *D. sim*, *D. simulans*; *D. sec*, *D. sechellia*. (**B**) Precursor sequences of miRNAs from the miR-982s cluster. Mature (miR) and miR* sequences are indicated in yellow and gray, respectively. When there is no annotation of miR* in the database, we define miR* as starting with 1 nt 3′ overhangs on the opposite arm of miR.

The miR-982s is X-linked, comprising five distinct miRNA families: miR-982, -2582, -303, -983 and -984. With the exception of the recently duplicated dme-miR-983-1/-2, miRNAs in this cluster do not share a seed sequence (**Fig. 2A &B**). Against the 12 *Drosophila* species [27], copies of this cluster can be found in *D.simulans*, *D. sechellia*, *D. yakuba* and *D. erecta* but are absent in all other more distantly related species. The expression of miR-982s members was confirmed by RT-PCR (**Fig. S1**). The evolution of this cluster in the *D. melanogaster* subgroup is depicted in detail in **Fig. 2A**.

**Figure 3:**
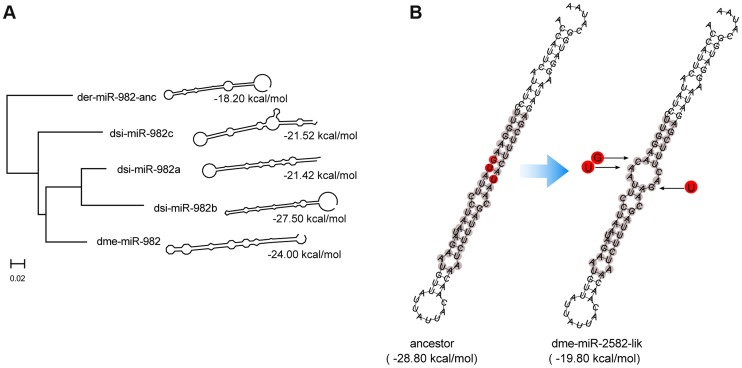
Evolution of the secondary structure of members of miR-982s as predicted by RNAfold [70]. (**A**) The thermo-stability of each hairpin is shown as kcal/mole at the tip of the branch. The phylogenetic tree is reconstructed based on the precursor sequences by the maximum likelihood method. (**B**) Disruption of dme-miR-2582-like hairpin structure by point mutations. Red nucleotide bases are the three lineage-specific mutations that disrupted the duplex. Gray nucleotide bases are the consensus miR:miR* duplex of miR-2582 inferred from dsi-miR-2582a/b.

As shown in **Fig. 2A**, each member of miR-982s appears to emerge *in situ* from local non-miRNA sequences. Due to their small sizes, unstructured genomic sequences evolving into miRNA-like transcripts have often been suggested [28] but have not been convincingly proven. The cluster of miR-982/2582/303/983/984 appears to be a good example of *de novo* origin (see below) with point mutations improving miRNA processing step by step (**Fig. 2B** **and Fig. S2 and S3**). For example, the secondary structure of miR-982 in *D. erecta* can only form a poor hairpin (−18.20 kcal/mol). Many nucleotide substitutions, accumulated subsequently in the stem and loop regions, have greatly improved the thermodynamic stability of the hairpin in *D.melanogaster* (−24.00 kcal/mol) and in the three paralogs of *D. simulans* (−21.52 to −27.50 kcal/mol; **Fig. 3A** **and Fig. S2 and S3**).

After each miRNA emerges from the unstructured sequence, gene duplication appears common [29], [30]. miR-2582 and miR-982 were expanded by whole-gene (**Fig. 2A**, Duplication 1, 2 and 3) or segment duplication (Duplication 4) in *D. melanogaster* and *D. simulans*, followed by gene conversion in *D. sechellia* (**Fig. 2A**). Moreover, miR-983 was duplicated in *D. melanogaster* (Duplication 5). In this species alone, miR-984 emerged *de novo* next to miR-983 (**Fig. 2A**).

These duplicates soon accumulated many nucleotide substitutions (**Fig. 2B**). Meanwhile, seed shifting and arm switching occurred in the miR-982/2582/303/983 families (**Fig. 2B**). These modifications presumably lead to new targets, resulting in neo-functionalization after gene duplication [28].

### II. Early adaptive evolution of new miRNAs

After new miRNAs emerged *de novo*, the question is whether the subsequent evolution is driven by natural selection. A greater level of divergence in miRNA genes than in flanking regions might suggest positive selection (Ref. [31]; **Fig. S4A**). A proper analysis would require the comparison of between-species divergence (D) and within-species polymorphism (P) using a modified McDonald-Kreitman (MK) test [32].

In this study, we generated DNA sequences from 42 *D. melanogaster* (∼7.5 kb from each line) and 25 *D. simulans* lines (∼8.1 kb) (**Table S4**). The D/P ratios for each precursor miRNA from miR-982s, as well as the 1 kb upstream flanking regions, were compared [33]. As shown in **Table 2**, all the miRNA genes from the miR-982, miR-303 and miR-983 families have a significantly higher D/P ratio than the flanking regions in both *D. melanogaster* and *D. simulans* (*p*<0.05), suggesting positive selection. Members of the miR-2582 family show significantly higher D/P ratios in *D. melanogaster*, but not in *D. simulans* (**Table 2**, also see next section).

**Table 2:** The McDonald-Kreitman test on individual miRNAs of the miR-982s cluster.

miRNA	D	P	D/P	p-value^d^
*D.melanogaster*				
dme-miR-982^a^	14	0	(n.a.)	6.31×10^−5^ ***
dme-miR-303	15	4	3.750	7.89×10^−3^ **
dme-miR-983	14	1	14.000	4.63×10^−4^ ***
dme-miR-2582-like	14	1	14.000	4.63×10^−4^ ***
Neighboring sites	31	38	0.816	
*D.simulans*				
dsi-miR-982c^b^	16	1	16.000	0.012*
dsi-miR-2582b^c^	11	5	2.200	0.468
dsi-miR-982b^b^	19	3	6.333	0.042*
dsi-miR-2582a^c^	11	6	1.833	0.579
dsi-miR-982a^b^	16	1	16.000	0.012*
dsi-miR-303	18	1	18.000	7.11×10^−3^ **
dsi-miR-983	17	1	17.000	9.39×10^−3^ **
neighboring sites	31	18	1.722	

D (for divergence) is the number of fixed differences between *D.melanogaster* and *D.simulans* and P is the number of polymorphic sites (P) within species.

a The consensus sequence of dsi-miR-982a/b/c is used as the outgroup of dme-miR-982.

b dme-miR-982 is used as the outgroup of dsi-miR-982a/b/c.

c dme-miR-2582-like is used as the outgroup of dsi-miR-2582a/b.

d Fisher's exact test was performed against neighboring site.

* , *p*<0.05;

** *p*<0.01;

*** *p*<0.001.

Because each individual miRNA gene, being small, would yield a significant result in the MK test only when the selection is extremely strong, we also performed the test on new miRNAs collectively, relative to the genome-wide 4-fold degenerate sites (from *Drosophila* Population Genomics Project (DPGP); see **Materials and Methods**). **Table 3** shows that the new miRNAs emerging in the last 30 Myrs have a higher D/P ratio than in the genome-wide 4-fold degenerate sites. In fact, more than 79% of the observed divergence in the precursors and more than 89% in the mature regions is estimated to have been fixed adaptively (see **Materials and Methods** and **Table 3**). A higher D/P ratio could also be attributed to an increase in selective constraint, rather than positive selection [34]. However, we excluded such possibility in **Text S1**. Due to the large number of adaptive sites, every new miRNA is likely to carry one or more of them. As expected, signatures of positive selection are much weaker for the lowly expressed miRNAs and mirtrons (**Table S5**).

**Table 3:** The McDonald-Kreitman test on the entire group of miRNAs of the same age.

site type		site number	D	P	P_DAF>5%_	D/P_DAF>5%_	MK test *p*-value^a^	α^b^ (% of adaptive fixations)
4-fold degenerate sites		3,495,672	378,361	168,979	83,996	4.50	-	-
0–4 Myrs	precursor	1,118	86	8	4	21.50	1.1e-4	79.1
	mature	196	18	0	0	Inf.	2.7e-2	100.0
4–30 Myrs	precursor	944	110	9	3	36.67	4.2e-7	84.7
	mature	227	33	3	0	Inf.	1.3e-3	89.8
30–60 Myrs	precursor	800	35	19	12	2.92	>0.50	−38.9
	mature	201	9	4	3	3.00	>0.50	−24.7
60–250 Myrs	precursor	2,839	110	37	14	7.54	2.6e-2	36.0
	mature	724	7	5	0	Inf.	0.25	19.6
>250 Myrs	precursor	6,335	72	60	18	4.00	>0.50	−49.8
	mature	1,470	2	3	0	Inf.	>0.50	−68.8

a One-tailed Fisher's exact test. To increase statistical power, we used polymorphisms with DAF>5% in the MK test [79].

b α was calculated using the methods described by Mackay *et al.* [75].

Other lines of evidence for recent adaptive evolution include the pattern of polymorphism within species and the differentiation between populations. The miR-982 cluster was examined further by the sliding window analysis of Fay and Wu's H (θ*_H_*), an estimator of nucleotide diversity sensitive to positive selection [35], [36]. The profile of θ*_H_* peaks near miR-983/984 and miR-303 in both species, a common footprint of hitchhiking with positive selection [35]. The signature is stronger in *D. simulans* for miR-982 than in *D. melanogaster* (**Fig. S4B and S4C**). In addition, we analyzed the M and Z populations of *D. melanogaster* [37]–[39] using the Fst statistic [40]. For dme-miR-984 and dme-miR-303, the precursor sequences are strongly differentiated between M and Z lines (Fst = 0.318 for dme-miR-984 and Fst = 0.252 for dme-miR-303) compared to all SNPs within the miR-982s region (Mann-Whitney U test, *p* = 0.057 for dme-miR-984 and *p* = 0.068 for dme-miR-303, **Table S6**) or the 238 *D. melanogaster* miRNAs (Mann-Whitney U test, *p* = 0.046 for dme-miR-984 and *p* = 0.008 for dme-miR-303; data were obtained from DPGP2 [41], see **Materials and Methods**). The analyses collectively suggest that the rapid evolution of new miRNAs is driven by natural selection.

### III. Death vs. integration after the initial adaptive evolution

After the initial adaptive evolution, one might reasonably expect these new adaptive miRNAs to be integrated into the transcriptional network and begin evolving at a slower rate. Surprisingly, the most likely fate of these new miRNAs was death, rather than integration. This can be seen in the number of observable new miRNAs from two different time periods – 22 surviving miRNAs from the last 30 Myrs but only 9 from the preceding 30 Myrs (30–60 Myrs before present).

**Figure 4:**
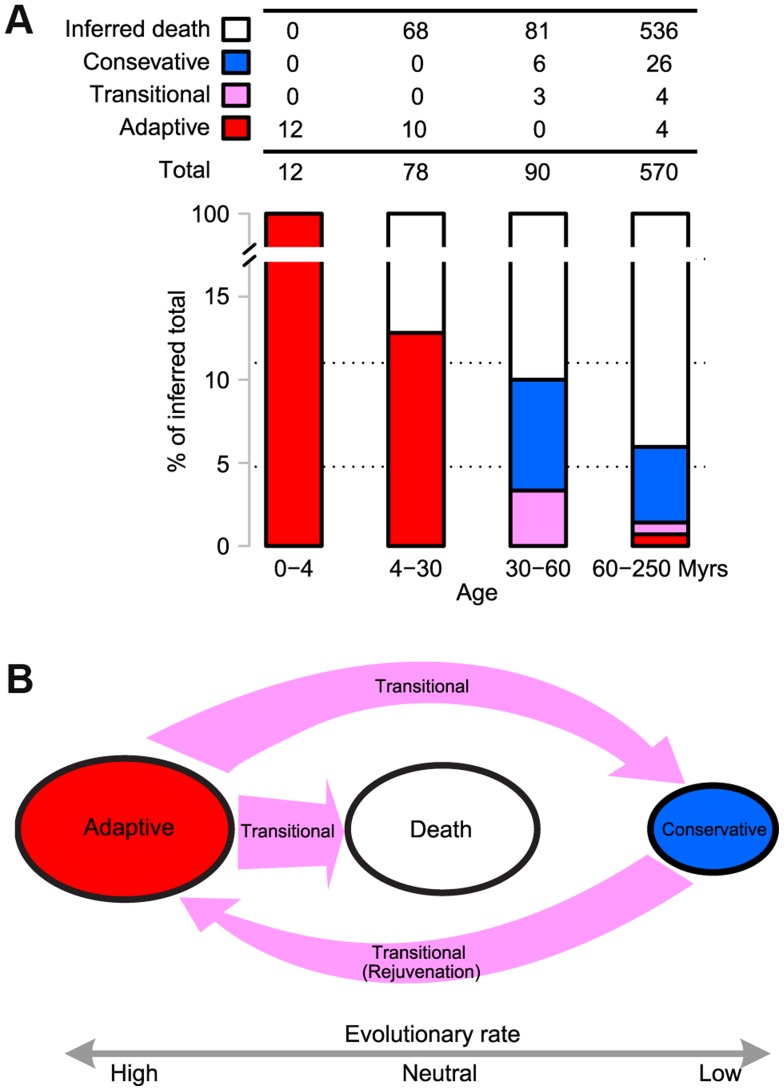
(**A**) **Evolutionary trajectories of miRNAs - The miRNAs were grouped by age as defined in** **Fig. 1** **.** The number of newborns in each time interval was estimated by assuming a constant birth rate obtained from the last 4 Myrs (3 miRNAs per Myr). The number of miRNA death was calculated by subtracting the observed number of surviving miRNAs from the inferred number of newborns (the birth rate multiplied by the time interval). The criteria for determining adaptive, conservative and transitional miRNAs are given in **Materials and Methods**. The number in each category is given in the table and the proportion is shown as a barplot. Note that the combined proportion of adaptive and transitional miRNAs, indicated in red and purple in the barplot, respectively, decreases chronologically. (**B**) **A model for the evolution of new miRNAs, which starts in the adaptive phase and ends in either death or in conservation.** In the latter phase, they may be recycled back to the adaptive phase. The evolutionary rate in each phase is indicated below. Transitions between phases are shown by arrows, the sizes of which reflect the flux between phases.

By assuming a constant birth rate, we can estimate the number of newborn miRNAs in each time interval, which can then be compared with the surviving miRNAs from that time period. Using the estimated rate of 3 newborn miRNAs per Myr (12 in the last 4 Myrs), **Figure 4A** shows that 87% of new miRNAs disappeared in 4–30 Myrs (68 out of 78). The proportion of death in older miRNAs increased only marginally, to 90%, for the period of 30–60 Myrs (81 out of 90). Therefore, most miRNAs seem to die quickly at an early stage of evolution, soon after the initial adaptive evolution. Only 6.0% of new miRNAs (34 out of 570) survived after 60 Myrs. It is unexpected that new adaptive miRNAs favored by natural selection should suffer such quick and massive death, albeit at a somewhat lower rate than neutrally evolving new miRNAs [12]. The former has a survival rate of 6.0% while the latter has a lower rate, at 2.5% [12]. Apparently, the initial adaptation is evolutionarily transient and the continual adaptation toward integration is not a common fate. We should note that alternative explanations have been considered. A most obvious one concerns the possibility of a bust of adaptive new miRNAs in *D. melanogaster* since its split from *D. simulans*. These explanations are compared in Discussion.

Interestingly, miRNA death may sometimes be an adaptive process. The miR-2582-like gene in *D. melanogaster* is shown to be evolving adaptively in **Table 2**, but its evolution is toward degeneracy. Three lineage-specific mutations that disrupt the duplex structure are shown in **Fig. 3B**, probably associated with the degeneration of dme-miR-2582. Presumably, conditions changed causing the adaptive function initially performed by the new miRNA to become deleterious at a later time.

Upon survival, new miRNAs eventually became integrated into the transcriptional network and evolved conservatively. There is a transitional phase after the adaptive phase, but before either integration or death. During the transition, these miRNAs often appeared to have a neutral evolutionary rate. **Figure 4A** shows that all the surviving miRNAs began to evolve either neutrally or conservatively (three transitional and six conservative miRNAs, respectively) within 30–60 Myrs (See **Materials and Methods**). The miR-2582 gene in *D. simulans* appears to be in such a transition (**Table 2**). It is interesting that miR-2582 orthologs in sibling species may be at different stages of evolution.

### IV. Cycles of adaptive-conservative evolution

Over long periods of time, new miRNAs will have died or have been integrated into the transcriptional network and are now conservatively evolving. miRNAs born 60–250 Myrs ago have largely vanished (94% have disappeared, see **Fig. 4A**). However, some of the cohort of the 34 surviving miRNAs are not behaving as expected. In fact, only 26 of them are evolving conservatively. Nearly a quarter of them (8 out of 34) are evolving either neutrally or adaptively (**Fig. 4A**) and most of these (7 out of 8) come from miR-972s or miR-310s (**Table 4**). At this rate of evolution, none of them should have been recognizable as homologs between *D. melanogaster* and *D. virilis*.

**Table 4:** K_miR_/K_S_ of the older miRNAs (60–250 Myrs) that have been evolving rapidly between *D. melanogaster* and *D. simulans*.

miRNA	K_miR_/K_S_ (*D. melanogaster* vs. *D. simulans*)	K_miR_/K_S_ (*D. melanogaster* vs. *D. virilis*)	Ratio
dme-miR-973^a^	1.152	0.264	4.4
dme-miR-974^a^	0.803	0.283	2.8
dme-miR-975^a^	0.571	0.389	1.5
dme-miR-976^a^	0.677	0.279	2.4
dme-miR-977^a^	1.096	0.616	1.8
dme-miR-311^b^	0.514	0.323	1.6
dme-miR-313^b^	0.877	0.238	3.7
dme-miR-964	0.898	0.303	3.0
mean	0.825	0.337	2.5

a miRNAs from miR-972s.

b miRNAs from miR-310s.

We suggest that the 8 unusual miRNAs may have been conservatively evolving for most of their evolutionary history. Four of them have been adaptively evolving once again and the remaining four appear to be in transition, away from the previous selective constraints. If the hypothesis is correct, we expect to see stronger evolutionary conservation in more distant comparisons than in recent ones. We use K_miR_/K_S_, where K_miR_ denotes the divergence in the precursor region of the miRNA, to measure conservation. **Table 4** shows their K_miR_/K_S_ values for the last 4 Myrs and for the distant past (60 Myrs after the split between *D. melanogaster* and *D. virilis*). The evolutionary conservation has indeed been relaxed substantially in the last 4 Myrs with the average value increasing from 0.337 to 0.825, a 2.5-fold difference. Such fold-changes of K_miR_/K_S_ were significantly high in the eight miRNAs, compared with the whole repertoire of 238 miRNAs (Mann-Whitney U test, *p* = 0.00014). The rate increase appears to be true in both *D. melanogaster* and *D. simulans* lineages when the homologous sequences from *D. yakuba* and *D. erecta* were used as outgroups to calculate the rate in each lineage separately. Among the eight genes, two and six are evolving slightly faster in *D. melanogaster* and *D. simulans*, respectively (see **Table S7**). It is interesting that some old miRNAs go through the reverse transition (or rejuvenation) from conservative to adaptive evolution, the latter being the hallmark of young miRNAs.

Rejuvenation can also lead to the death of old miRNAs. The miR-972s may be such an example. Some members of this cluster emerged 60–250 Myrs ago and should have been integrated into the ancestral genome by the time *D. pseudoobscura* split from *D. melanogaster*. However, the entire miR-972s region was lost in *D. pseudoobscura* since the split.

Taken together, new miRNAs (such as miR-310s and miR-972s) may go through cycles of adaptation, integration (if escaping death) and rejuvenation, which would start another cycle of adaptation and integration (**Fig. 4B**).

### V. Evolution of miRNA expression

**Figure 5:**
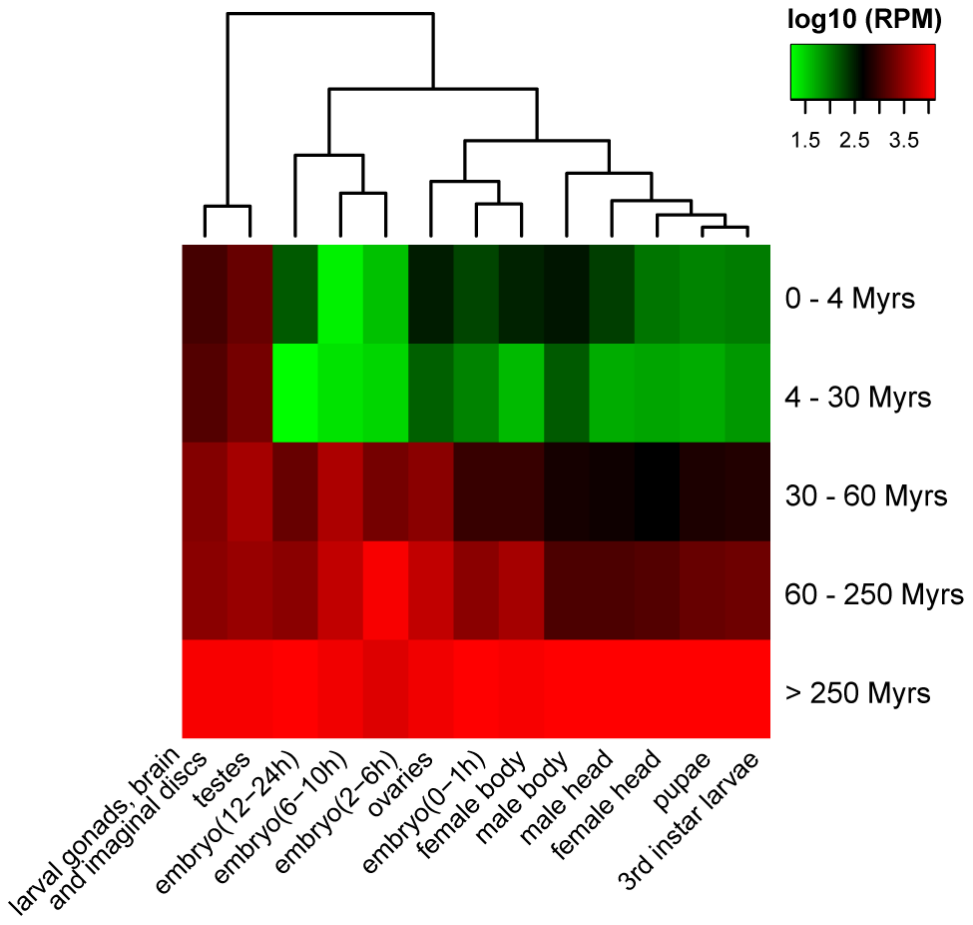
Clustering of miRNA expression among different age groups and tissues (or development stages). Expression level of each miRNA age group is calculated based on small RNA libraries from different tissues and developmental stages of *D. melanogaster* (**Table S1**).

To study the evolution of new miRNAs sequences, we characterized their expression patterns. We did so by using the global small RNA profiling datasets (see **Table S1** and **Materials and Methods**). **Figure 5** shows young miRNAs (<30 Myrs) are lowly expressed in specific tissues, generally in the testes and larval brain/gonads/imaginal discs. Middle-aged miRNAs (30–60 Myrs) broadened their expressions to include ovaries and embryos. The older miRNAs (60–250 Myrs) showed moderate and even broader expressions, which then evolved to become highly abundant in all tissues and developmental stages as seen in the oldest miRNAs (>250 Myrs). The simplest explanation is that new miRNAs increase the expression level and expand the breadth as they get older. The change in expression parallels that in sequence evolution (**Fig. 4A** and **Table S8**). There are other explanations that may also account for the different expression patterns between new and old miRNAs (see **Text S2**). Detailed descriptions of the evolution in expression patterns are given in **Text S3**.

## Discussion

During Metazoan evolution, the miRNA repertoire expanded dramatically from a few genes to several hundreds [28], [42]. By limiting the analysis to new miRNAs that evolve adaptively soon after their birth, we avoided the large number of lowly expressed miRNA-like sequences. These sequences may or may not be considered miRNAs and are generally thought to be evolutionarily ephemeral and adaptively insignificant [43], [44]. The inclusion of only new miRNAs that evolve adaptively at emergence reveals an unexpected pattern of an excess of such miRNAs in the last 4 million years of the *D. melanogaster* lineage. The possible explanations are therefore either a burst of birth since *D. melanogaster* split from *D. simulans*, or a decline in the survivorship of adaptive new miRNAs as they age.

We consider the latter explanation as more plausible for several reasons. First, the birth rate of miRNA-like sequences indeed appears constant because different *Drosophila* species have comparable numbers of such new transcripts [16]. Given the ease in forming precursor-like hairpins, the constant rate is hardly surprising. Second, as a result, the birth rate of adaptive new miRNAs may not deviate much from a constant value either. Indeed, the burst of adaptive new miRNAs is observable in *D. simulans* as well as the common ancestor of *D. melanogaster* and *D. simulans*, as is evident in the miR-982 cluster (**Fig. 2A**). Third, the proportion of adaptive miRNAs born in the period of 4–30 Myrs is also higher than that in the 30–60 Myrs period. Overall, an excess of new adaptive miRNAs appears to be a decreasing function of time, rather than of particular lineages; hence, their death over time is a simpler explanation.

Because only a small number of new adaptive miRNAs remain active after cycles of evolution through phases of adaptation and degeneration, the repertoire of miRNAs in the *D. melanogaster* genome has been nearly static in 40 Myrs of evolution, with only 0.18 miRNA integrations per Myrs. We should note that this low rate may still be an over-estimate because not all death has been accounted for. This (near) steady state echoes the view of a correlation between morphological complexity and the size of miRNA repertoire [45], as the *Drosophila* genus has been relatively invariant in form since its diversification.

Despite the low integration rate, many new miRNAs continue to emerge and some briefly evolve adaptively before their demise. This “transient utility” is puzzling as gene functions are lost usually through environmental changes (such as vision genes in caves [46]) or redundancies [47]. A possible explanation may be the suggested role of miRNAs in evolutionary canalization [7]. In such a role, the regulators and their targets need not be stringently wired as long as the system remains properly buffered. By this scheme, new miRNAs may emerge to fill in the transiently vacated role created by the shifting interactions between established miRNAs and their targets [7]. They disappear when the role is no longer needed.

A small number of new miRNAs that become integrated into the transcriptional network begin this process in the testis, in parallel with new protein coding genes [48]–[54]. Since sexual selection driving male reproduction is a very potent force of evolution, this expression pattern may not be all that surprising [50], [55]–[58]. In the example of miR-982s, the predicted targets are indeed enriched in genes of male courtship behavior and other male sexual traits (**Table S9**). Once a new miRNA is established, its expression is often broadened to other tissues. Testis may be the beachhead that permits the new miRNA to gradually modulate its expression and interactions with potential targets. In addition, new miRNAs with distinct seeds often emerge in clusters, which presumably facilitate their co-expression [29], [30], [59].

Unlike protein coding genes, miRNAs can easily emerge *de novo*, thanks to their small size, but can often be derived from existing genes as well [60]. The simple structure of miRNAs may permit general inferences on features and dynamics of genic evolution. A previous example is the rate of evolution as a correlate of expression level [61]. It would be interesting to see if the inferred cycles of evolution experienced by new miRNAs are a general process.

## Materials and Methods

### Sample RNA library preparation and sequencing

Total RNA was extracted from *D. simulans* (NC48S) and from *D. pseudoobscura* using TRIzol (Ambion). Ovaries and testes from 3 to 5-day adults were dissected and collected for both NC48S and *D. pseudoobscura*. Imaginal discs including central nerve system (CNS) were dissected from wandering third-instar larva of *D. pseudoobscura*. Small RNA libraries were generated from each RNA sample using Illumina Small RNA Sample Preparation kit, and sequenced with the Illumina HiSeq 2000 at the Beijing Genomics Institute (Shenzhen). The data were deposit at Gene Expression Omnibus (GEO) database (http://www.ncbi.nlm.nih.gov/geo/) under the accession numbers GSM1165052-GSM1165056.

### Data compilation

The publicly available small RNA sequencing reads from four *Drosophila* species (*D. melanogaster*, *D. simulans*, *D. pseudoobscura* and *D. virilis*) were downloaded from GEO database (http://www.ncbi.nlm.nih.gov/geo/, **Table S1**). The miRNA sequences of three *Culicinae* species (*Aedes albopictus*, *A. aegypti* and *Culex quinquefasiatus*) were adopted from two previous small RNA sequencing studies [24], [25]. *Drosophila* genome sequences were retrieved from UCSC (http://genome.ucsc.edu); the Whole Genome Alignment (WGA) and CDS alignment were obtained from 12 *Drosophila* Assembly/Alignment/Annotation (http://rana.lbl.gov/drosophila). The genome versions used were: *D. melanogaster*, dm3; *D. simulans*, droSim1; *D. sechellia*, droSec1; *D. yakuba*, droYak2; *D. erecta*, droEre2; *D. ananassae*, droAna3; *D. pseudoobscura*, dp4; *D. persimilis*, droPer1; *D. willistoni*, droWil1; *D. mojavensis*, droMoj3; *D. virilis*, droVir3; *D. grimshawi*, droGri2. The genome coordinates and sequences of miRNA genes were retrieved from miRBase Release 19 (http://www.mirbase.org). The genome coordinates and sequences of intron, rRNA, tRNA, snRNA and transposon elements were obtained from FlyBase (r5.41, http://flybase.org,)

### Defining canonical miRNAs, mirtrons and miRNA clusters

We defined canonical miRNAs and mirtrons according to Ruby *et al.* [62]. Mirtrons were defined as pre-miRNAs with both 5′ and 3′ ends matching the splicing sites of host introns. The rest of the miRNAs were then classified as canonical miRNAs. When more than three miRNAs were located within a 20 kb region, these miRNAs were considered as a cluster.

### miRNA annotation and expression analysis

Small RNA reads (18–30 nt) were extracted from sequencing data. Firstly, we excluded reads mapped to transposon elements and structural RNAs (rRNA, tRNA and snRNA) using bowtie [63], allowing no mismatch. Next, we annotated novel miRNAs by miRDeep2 [64] with default parameters. Finally, miRNAs with no read matching miR* were removed following previous practice [23]. We combined novel miRNAs sequences and known miRNA sequences for expression analysis.

For each species, small RNA reads (18–30 nt) were mapped to miRNA precursor sequences using bowtie [63], allowing no mismatch. Each read count was divided by the number of matches to miRNA precursors. The miRNA expression was normalized by total miRNA counts and scaled to reads per million (RPM), as previous described [18].

### Phylogenetic dating of miRNAs

We examined phylogenetic distributions of the *D. melanogaster* miRNAs in three other *Drosophila* species (*D. simulans*, *D. pseudoobscura* and *D. virilis*) and three *Culicinae* species (*Aedes albopictus*, *A. aegypti* and *Culex quinquefasiatus*), where small RNAs have been profiled via deep sequencing [12], [16], [22], [24], [25]. Based on the comprehensive dataset, miRNA homologs were determined by homology search using either the whole genome alignment (WGA) within the *Drosophila* group or BLAST (threshold E<10^−5^) between *Drosophila* species and mosquitoes, and cross-checked with small RNA reads in the species in query (at least one read matching mature and miR*).

The homologous sequences of the *D. melanogaster* miRNA precursors in *D. simulans* (droSim1), *D. pseudoobscura* (dp4) and *D. virilis* (droVir3) were extracted from UCSC pairwise WGAs using LiftOver (http://hgdownload.cse.ucsc.edu/, minMatch = 0.6). The precursors failing to obtain hits in the genomes were subjected to BLASTN search against NCBI trace archives (http://www.ncbi.nlm.nih.gov/Traces/home/). Matched sequences with E-values <10^−5^ were also considered as miRNA homologs and recovered for the analysis below. The WGA output was compared with miRNA annotation by miRDeep2 [64]; miRNA orthologs confirmed by miRDeep2 were retained.

The miRNA precursor sequences in *Aedes albopictus*, *Culex quinquefasiatus* and *A. aegypti* were adopted from the studies of Li *et al.* [24] and Skalsky *et al.* [25]. These sequences were combined and subjected to BLASTN search against miRNA precursors in *D. melanogaster*. The best reciprocal hits with E-values <10^−5^ were retained as the corresponding miRNA homologs in the *Culicinae* lineage.

According to the phylogenetic distribution, maximum parsimony method was used to infer the origination of each miRNA along the main trunk of the phylogenetic tree of *D.melanogaster*, *D. simulans*, *D. pseudoobscura*, *D. virilis* and *Culicinae*. An miRNA is assumed to emerge in the most recent common ancestor of all the species bearing the authentic homologs. The branch lengths of the phylogenetic tree (in Myrs) were adopted from previous estimations [27], [65], [66]. The 238 miRNAs were classified into five age groups, corresponding to the time intervals of 0–4 Myrs, 4–30 Myrs, 30–60 Myrs, 60–250 Myrs and >250 Myrs.

### Genealogy of miR-982s in *Drosophila* species

The genomic coordinates and precursor sequences of dme-miR-982/303/983-1/983-2/984 and dsi-miR-982c/2582b/982b/2582a/982a/303/983 were retrieved from miRBase (Release v19). Based on the WGA of 12 *Drosophila* genomes [27], genomic sequence of the whole miR-982s cluster (∼9 Kb) in *D. melanogaster* (dm3) was extracted and used as a query to search against the other 11 *Drosophila* genomes using BLAT [67] with an E-value threshold of 0.001. We only detected hits in *D. simulans*, *D. sechellia*, *D. yakuba* and *D. erecta*, indicating that miR-982s is specific to the *melanogaster* subgroup. Homologous sequences of the miR-982s cluster from the five species were aligned using MUSCLE [68]. Homologs of miR-982s members in each species were identified using BLAST with the query of known precursor sequences (miRBase Release v19) and an E-value threshold of 0.001. The hits were further inspected in the alignment of the whole miR-982s cluster. The phylogenetic tree of each family of miR-982, miR-2582, miR-303, and miR-983 was reconstructed using the maximum likelihood method as implemented in MEGA 5.0 [69].

To validate the existence of miR-982s members in *D. yakuba* and *D. erecta*, we first predicted the secondary structure and thermo-stability of each miRNA homolog using RNAfold (http://rna.tbi.univie.ac.at/) with the default parameters [70]. A good hairpin with minimum free energy (MFE) >15 kcal/mol was considered as a potential miRNA candidate. There were four such candidates: dya-miR-2582-anc, dya-miR-303-anc, der-miR-982-anc, and der-miR-983-anc, where “anc” indicates ancestor. Then, we validated the expression of each candidate by amplifying the potential miRNA precursor from cDNA because the mature miRNA is hard to define. Total RNAs were extracted from testes of *D. yakuba* and *D. erecta* using TRIzol (Ambion) and treated with TURBO DNase Kit (Ambion). 0.5 ug RNA was reverse transcribed (RT) in a 20 ul reaction volume using PrimeScript II 1st Strand cDNA Synthesis Kit (TaKaRa). 1 ul RT products were used for PCR with Ex Taq DNA Polymerase (TaKaRa). PCR primers used are listed in **Table S10**.

### Population genetic analysis of miR-982s in *D. melanogaster* and *D. simulans*

A total of 25 *D.simulans* lines and 42 *D.melanogaster* lines, including 29 M lines and 13 Z lines, were used for population sequencing of the miR-982s cluster. The fly strains used were listed in **Table S4**. The genomic sequences of *D.simulans* (droSim1) and *D.melanogaster* (dm3) were used to design primer pairs that amplify a ∼8 Kb region spanning the whole miR-982s cluster and ∼1.5 Kb each of the upstream and downstream flanking regions. The PCR product of each primer set was designed to be about 2 Kb in length and overlapped with each other by at least 300 bp. The primers used are listed in **Table S10** and their genomic coordinates are displayed in **Fig. S5**. PCR was carried out using LA Taq DNA Polymerase (TaKaRa). PCR products were subject to direct sequencing or clone sequencing on an ABI 3730xl DNA Analyzer (Applied Biosystems). DNA sequences were assembled using SeqMan software (DNASTAR Inc., USA) and aligned using MUSCLE [68] with manual inspection. Haplotypes were inferred with the PHASE program when heterozygous sites were present [71]. The sequences obtained in this study have been deposited in GenBank under the accession numbers JX648211-JX648278.

Using the population sequencing data, several methods were used to detect positive selection of miR-982s in *D. melanogaster* and *D.simulans*, respectively. First, MK tests were applied on each member of miR-982s based on the divergence between *D. melanogaster* and *D.simulans* consensus sequences and polymorphism within either species. Each miRNA precursor was tested against a 1 kb region about 1.5 kb upstream of the 5′ end of miR-982s. Second, sliding window analysis of divergence and polymorphism was applied to the whole miR-982s cluster and its flanking region. The divergence was calculated using Kimura's 2-parameter model [72] based on the genomic sequences of *D. simulans* (droSim1) and *D. melanogaster* (dm3). The polymorphism within either species was estimated using the method described previously [35], [36], [73], [74]. *D. simulans* (droSim1) and *D. melanogaster* (dm3) were used as the outgroup for each other reciprocally, in order to polarize the derived alleles. The window size is 100 bp and the step width is 25 bp. Finally, based on our miR-982s population data or DPGP2 data (see below) [41], the pattern of population differentiation (Fst) between Z and M lines was estimated for each miRNA precursor using Weir's method [40].

### Analysis of the evolutionary fate of miRNAs

We used the McDonald-Kreitman test (MK test) [32] framework to detect positive selection in miRNAs from each age group based on the polymorphisms within *D. melanogaster* and the divergence between *D. melanogaster* and *D. simulans*. Precursor or mature sequences of each miRNA group were combined and treated as the functional category, while the 4-fold degenerate sites in the whole genome were used as the neutral control. The divergence is calculated by counting the number of changed nucleotide sites between *D. melanogaster* (dm3) and *D. simulans* (droSim1) based on the UCSC whole genome alignment. Polymorphism data was retrieved from *Drosophila* Population Genomics Project (DPGP, http://www.dpgp.org/, release 1.0). SNPs that were detected on more than thirty individuals and exhibited a derived allele frequency (DAF) >5% were used for the MK test.

The proportion of adaptively fixed mutations (α) was estimated as previously described [75]. To estimate the evolutionary fate of each miRNA, we first screened for adaptive miRNAs among the 238 candidates by using each miRNA's precursor together with the 50 bp flanking sequences on both sides as the functional sites. The *p*-values of multiple MK tests were adjusted by the Benjamini-Hochberg method [76] and the adaptive significance of each candidate is re-validated by using the precursor alone in the MK test. We then identified the conservative miRNAs by comparing the number of substitutions in the miRNA precursors (K_miR_) with the number of substitutions in the synonymous sites (K_S_) between *D.melanogaster* and *D.simulans*. miRNAs with K_miR_/K_S_<0.5 were considered to be conservatively evolving. Kimura's 2-parameter model [72] and the Nei-Gojobori model [77] were used to calculate K_miR_ and K_S_, respectively. Finally, excluding the adaptive and conservative miRNAs, the remaining were considered to be in transition between adaptive to conservative/death.

### Evolutionary analysis of miRNA expression patterns

Data processing of small RNA deep sequencing libraries from different development stages and tissues of *D. melanogaster* [12], [16], [18]–[21], [23] was conducted as described above. The read counts of each miRNAs were normalized to Reads Per Million (RPM), which is the read number of each miRNA per million mapped reads in each library. The normalized counts were log2 transformed and subject to hierarchical clustering using R package heatmap2.

### Target prediction of miR-982s and functional annotation

miR-982s targets were predicted by seed match using TargetScan (v5.0 http://www.targetscan.org/fly_12/) [5]. Taking all the miRNA members together, 1,002 targets were obtained in *D. melanogaster* and 3,563 in *D. simulans*, of which 454 were shared by both species. We used DAVID to perform a Gene Ontology (GO) enrichment test for the predicted targets in the two species (DAVID v6.7, http://david.abcc.ncifcrf.gov/) [78]. Only the GO terms for biological processes were used for the enrichment test.

## Supporting Information

Figure S1 The verification of the existence of miR-982s members in *D. yakuba* or *D. erecta*. (**A**) Prediction of secondary structures for the ancestral miRNA candidates. For miR-2582 and miR-303, homologous sequences in *D. yakuba* were used; for miR-982 and miR-983, homologous sequences in *D. erecta* were used. Minimum free energy is labeled below each precursor. (**B**) Gel analysis of potential expression of the candidate miRNA precursors in **Fig. S1A**. RT-PCR was conducted using total RNA extracted from testes of *D. yakuba* (for miR-2582/303) or *D.erecta* (for miR-982/983).
(PDF)

Figure S2 Multiple sequence alignment of miRNA families in miR-982s. (A) miR-2582 family, (B) miR-982 family, (C) miR-303 family and (D) miR-983 family. The consensus secondary structures were denoted above the alignments.
(PDF)

Figure S3 Genealogies and analysis of secondary structures of miR-982s. (A) miR-2582 family, (B) miR-982 family, (C) miR-303 family and (D) miR-983 family. Genealogical trees were constructed using maximum likelihood method implemented in MEGA 5.0 [69]. Secondary structures of homologus miRNA sequences were predicted using RNAfold (http://rna.tbi.univie.ac.at/) with the default parameters [70]. Estimated minimal free energy (MFE) is labeled for each hairpin. Color bar indicates base-pairing probabilities (or the probability of being unpaired in the unpaired regions).
(PDF)

Figure S4 Sliding window analysis of divergence and polymorphism of miR-982s (window size = 100, step = 25). Gene structures are illustrated below the window with orange blocks for miRNA genes, grey blocks for exons of *CG3626* and dash lines for alignment gaps. (**A**) Divergence between *D. melanogaster* and *D. simulans*. The upper and lower lines indicate gene structures of miR-982s in *D. melanogaster* and *D. simulans*, respectively. Orange blocks denote dme-miR-982, dme-miR-303, dme-miR-983-1, dme-miR-983-2 and dme-miR-984 in *D. melanogaster*, and dsi-miR-982c, dsi-miR-2582b, dsi-miR-982b, dsi-miR-2582a, dsi-miR-982a, dsi-miR-303 and dsi-miR-983 in *D. simulans*. (**B**) Polymorphism in *D. melanogaster*. Gene structure is indicated as the upper line in (**A**). (**C**) Polymorphism in *D. simulans*. Gene structure is indicated as the lower line in (**A**).
(PDF)

Figure S5 Primer design of miR-982s for PCR amplification and sequencing. Blue ticks denote the positions of primers. Red ticks denote miRNA genes.
(PDF)

Table S1 The GEO accession numbers of the small RNA libraries used in this study.
(PDF)

Table S2 Normalized miRNA expression (RPM) in different tissues and developmental stages of *D. melanogaster*. Small RNA sequencing data were retrieved from GEO database as listed in Table S1.
(XLSX)

Table S3 miRNAs clustered in miR-982s, miR-310s, and miR-972s in *D. melanogaster*.
(PDF)

Table S4 Fly strains used for population sequencing of the miR-982s cluster.
(PDF)

Table S5 Estimates of proportions of selection regimes on different classes of miRNA genes. Both canonical miRNAs and mirtrons from different age groups were classified into two subgroups according to their expression levels: low (≤200 RPM) and high (>200 PRM). The number of genes in each category is given in the parentheses. D, divergence between *D. melanogaster* and *D. simulans*; P, polymorphism in populations of *D. melanogaster* (DPGP); DAF, derived allele frequency; α, the fraction of adaptive fixations; b, the fraction of new mutants that are weakly deleterious; d, the fraction of new mutants that are strongly deleterious; f, the expected number of neutral segregating sites.
(PDF)

Table S6 Fst of each miRNA from miR-982s between M-line and Z-line.
(PDF)

Table S7 K_miR_/K_S_ of the older miRNAs (60–250 Myrs) that have been evolving rapidly between *D. melanogaster* and *D. simulans*. The common ancestral sequences of *D. melanogaster* and *D. simulans* are inferred from *D. yakuba* and *D. erecta*.
(PDF)

Table S8 Age and evolutionary mode of the 136 highly expressed, canonical miRNAs analyzed in this study.
(PDF)

Table S9 GO enrichment of the predicted target genes of miR-982s in *D. melanogaster* and *D. simulans*. Targets were predicted by seed match using TargetScan (v5.0 http://www.targetscan.org/fly_12/) [5].
(PDF)

Table S10 PCR primers of the miR-982s cluster.
(PDF)

Text S1 Interpretation of McDonald-Kreitman test result.
(PDF)

Text S2 An alternative explanation for expression evolution of new and old miRNAs.
(PDF)

Text S3 The evolution of miR-982s, miR-310s and miR-972s expression.
(PDF)
