# Supplementary figures and images for: New MicroRNAs in Drosophila—Birth, Death and Cycles of Adaptive Evolution
Source: PLoS Genet. 2014 Jan 23;10(1):e1004096. doi: 10.1371/journal.pgen.1004096 (PMC3900394; doi:10.1371/journal.pgen.1004096)

A

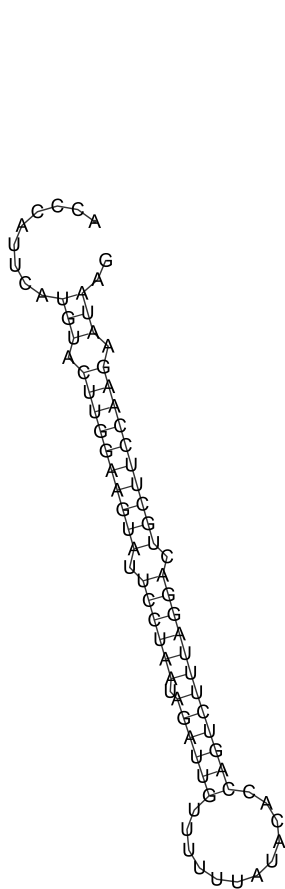

dya-mir-2582-anc  
-27.50 kcal/mol

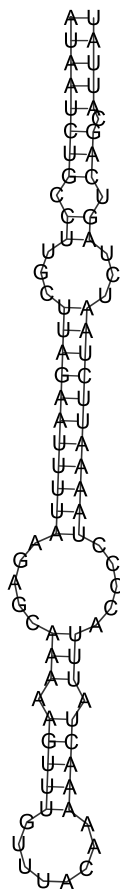

dya-mir-303-anc  
-16.50 kcal/mol

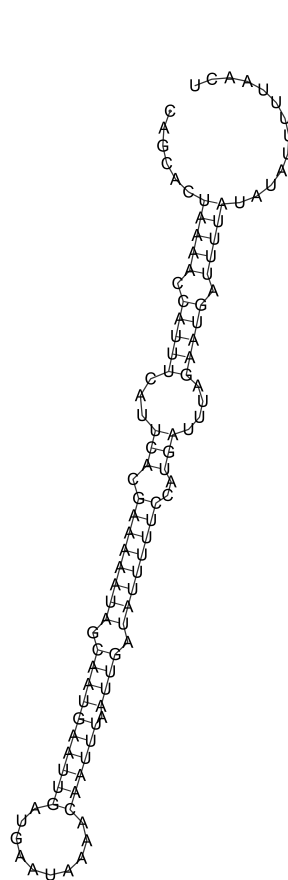

der-mir-982-anc  
-18.20 kcal/mol

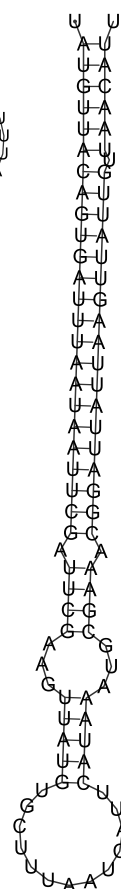

der-mir-983-anc  
-31.12 kcal/mol

B

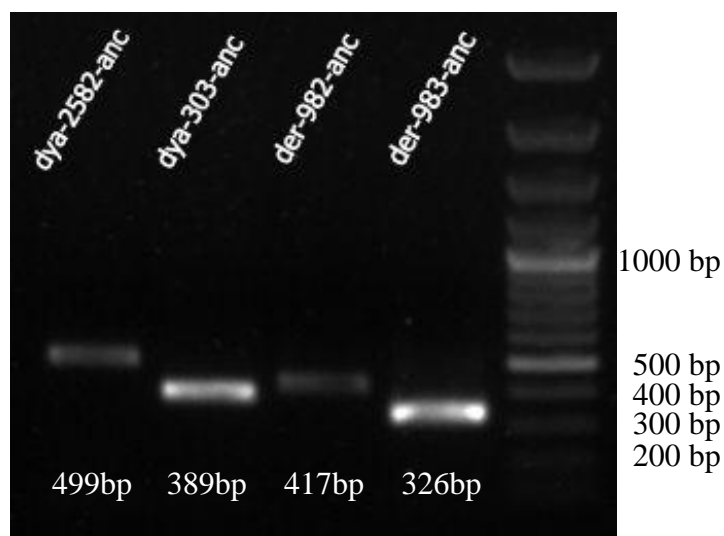

Supplement: Figure S1 — The verification of the existence of miR-982s members in D. yakuba or D. erecta. (A) Prediction of secondary structures for the ancestral miRNA candidates. For miR-2582 and miR-303, homologous sequences in D. yakuba were used; for miR-982 and miR-983, homologous sequences in D. erecta were used. Minimum free energy is labeled below each precursor. (B) Gel analysis of potential expression of the candidate miRNA precursors in Fig. S1A. RT-PCR was conducted using total RNA extracted from testes of D. yakuba (for miR-2582/303) or D.erecta (for miR-982/983). (PDF) [file pgen.1004096.s001.pdf]

A

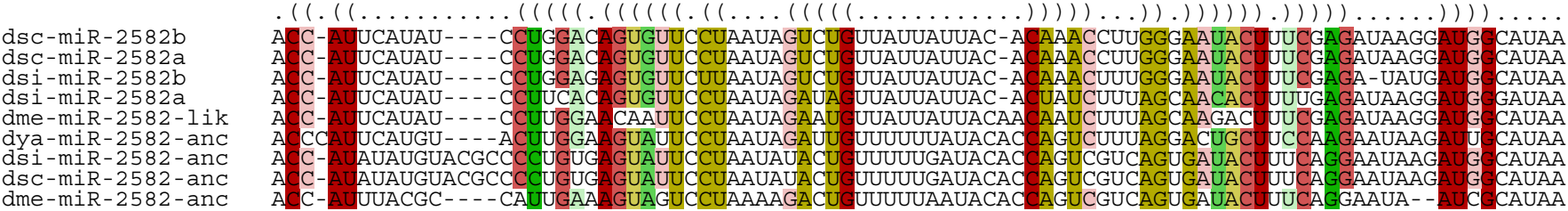

B

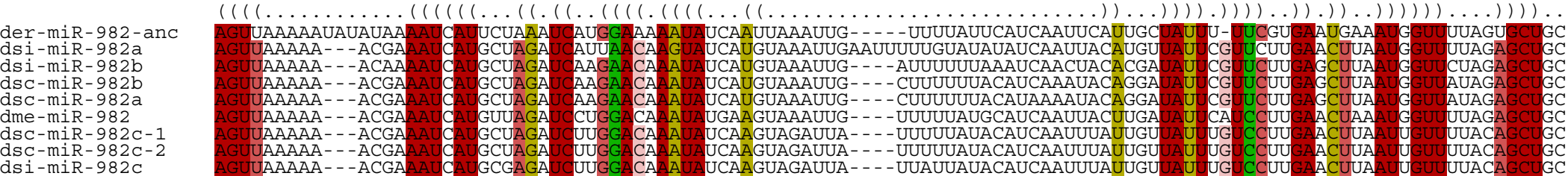

C

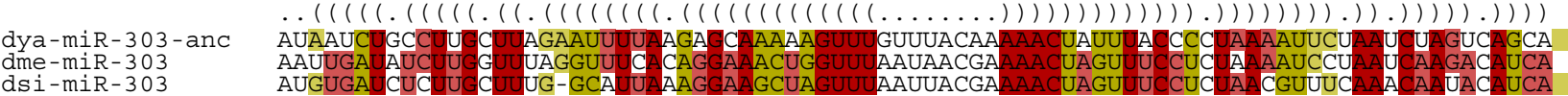

D

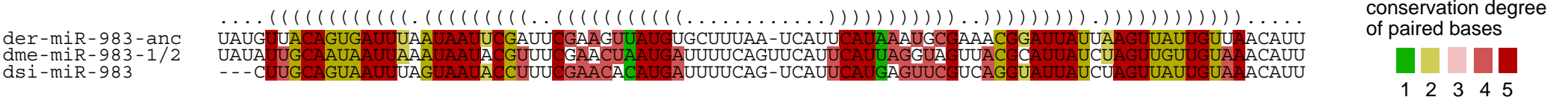

Supplement: Figure S2 — Multiple sequence alignment of miRNA families in miR-982s. (A) miR-2582 family, (B) miR-982 family, (C) miR-303 family and (D) miR-983 family. The consensus secondary structures were denoted above the alignments. (PDF) [file pgen.1004096.s002.pdf]

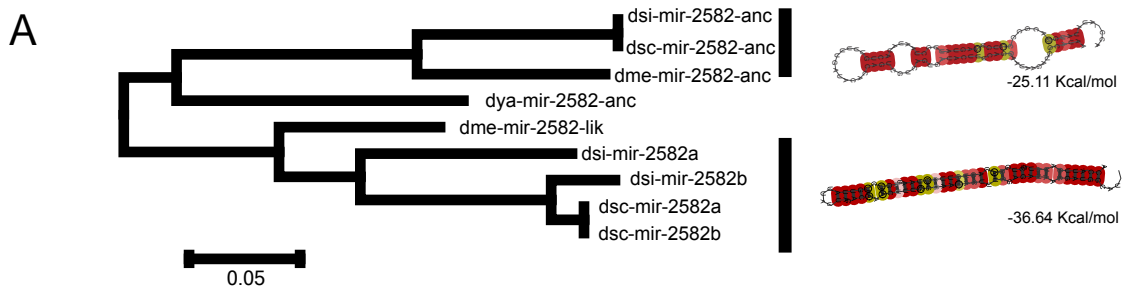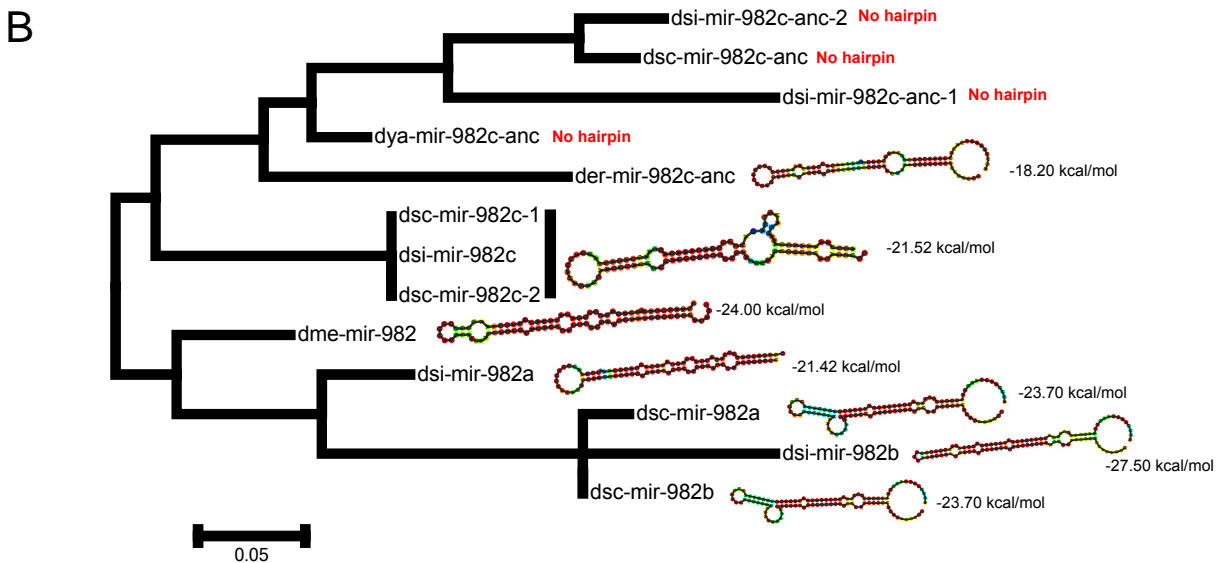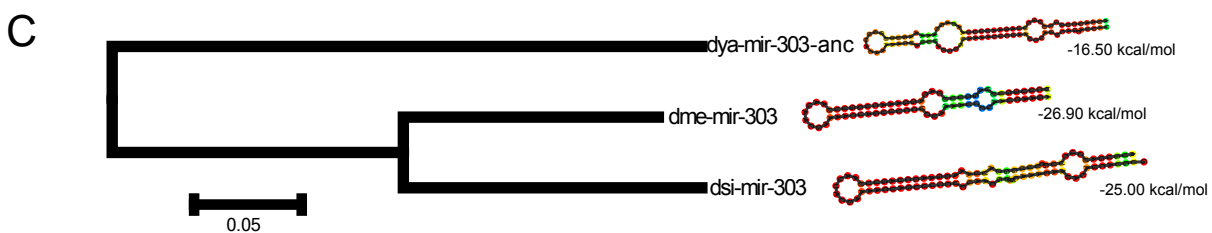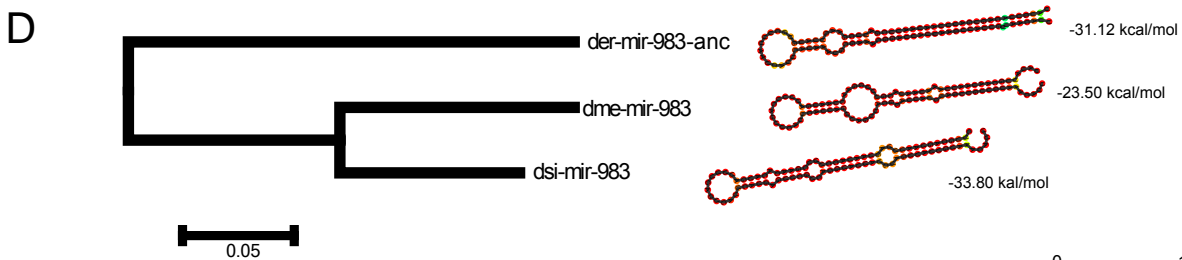

Supplement: Figure S3 — Genealogies and analysis of secondary structures of miR-982s. (A) miR-2582 family, (B) miR-982 family, (C) miR-303 family and (D) miR-983 family. Genealogical trees were constructed using maximum likelihood method implemented in MEGA 5.0 [69]. Secondary structures of homologus miRNA sequences were predicted using RNAfold (http://rna.tbi.univie.ac.at/) with the default parameters [70]. Estimated minimal free energy (MFE) is labeled for each hairpin. Color bar indicates base-pairing probabilities (or the probability of being unpaired in the unpaired regions). (PDF) [file pgen.1004096.s003.pdf]

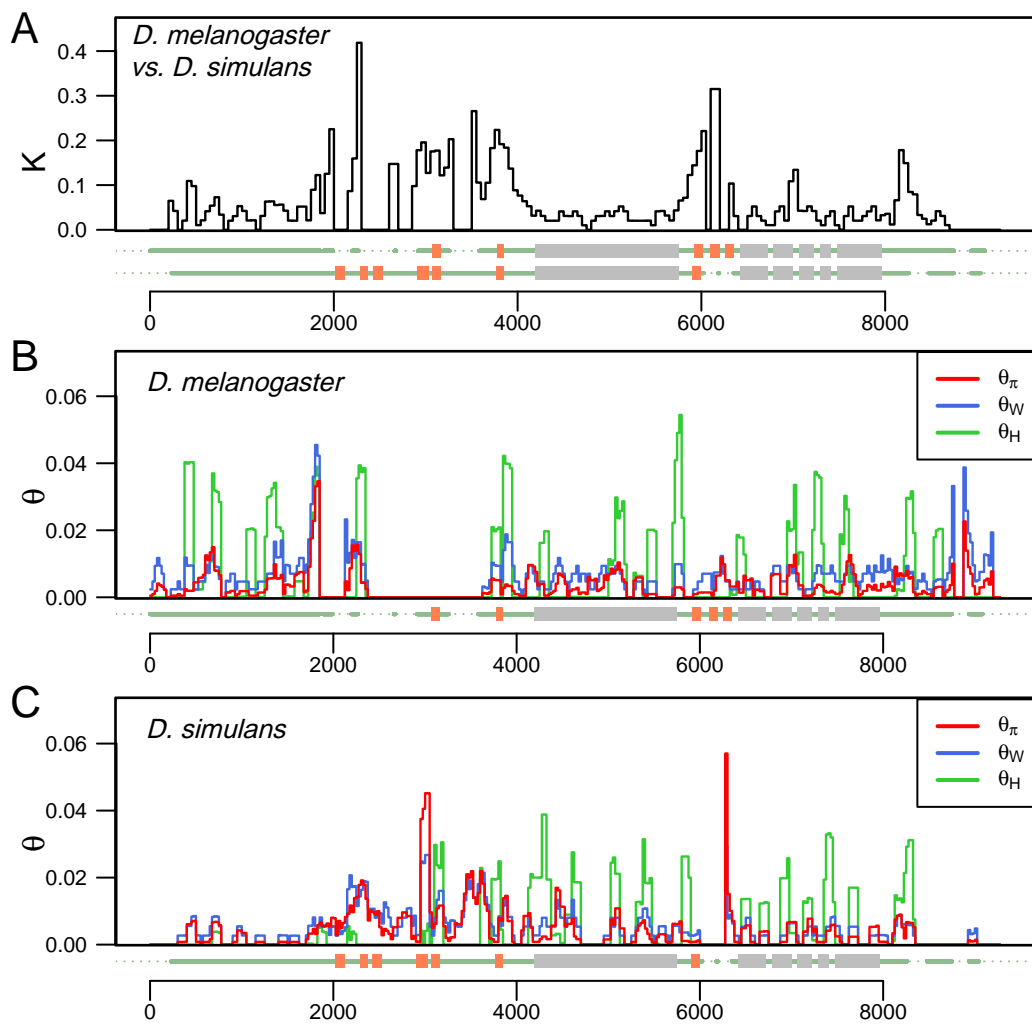

Supplement: Figure S4 — Sliding window analysis of divergence and polymorphism of miR-982s (window size = 100, step = 25). Gene structures are illustrated below the window with orange blocks for miRNA genes, grey blocks for exons of CG3626 and dash lines for alignment gaps. (A) Divergence between D. melanogaster and D. simulans. The upper and lower lines indicate gene structures of miR-982s in D. melanogaster and D. simulans, respectively. Orange blocks denote dme-miR-982, dme-miR-303, dme-miR-983-1, dme-miR-983-2 and dme-miR-984 in D. melanogaster, and dsi-miR-982c, dsi-miR-2582b, dsi-miR-982b, dsi-miR-2582a, dsi-miR-982a, dsi-miR-303 and dsi-miR-983 in D. simulans. (B) Polymorphism in D. melanogaster. Gene structure is indicated as the upper line in (A). (C) Polymorphism in D. simulans. Gene structure is indicated as the lower line in (A). (PDF) [file pgen.1004096.s004.pdf]
