# Supplementary material for: New MicroRNAs in Drosophila—Birth, Death and Cycles of Adaptive Evolution
Source: PLoS Genet. 2014 Jan 23;10(1):e1004096. doi: 10.1371/journal.pgen.1004096 (PMC3900394; doi:10.1371/journal.pgen.1004096)
Supplement: Figure S5 — Primer design of miR-982s for PCR amplification and sequencing. Blue ticks denote the positions of primers. Red ticks denote miRNA genes. (PDF) [file pgen.1004096.s005.pdf]

D. melanogaster

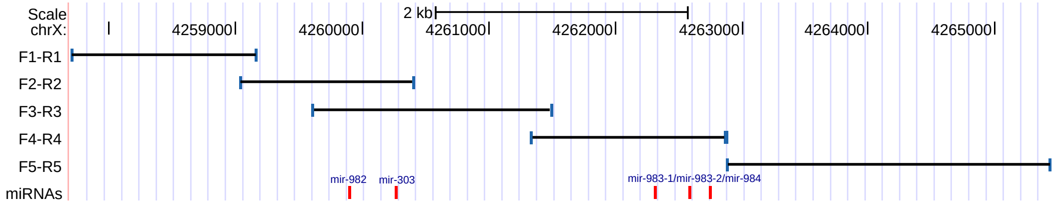

Primers

F1 TAACCAACGCCTACGACTAT
F2 AGCCTCATATCACCGCTGTC
F3 CAATGGCGTAAATGGTTGTG
F4 TGCCATCAAGAAGATTCACAGCG
F5 AAGCCATTCCCATTCACTCAC

R1 TCTAACATTTGGGCTCATTT
R2 GAAACTGCATTCCCCGAACG
R3 CGTGTGCAAATAGTGGTGCT
R4 CGCTGTGAATCTTCTTGATGGCA
R5 AGACACCGACCACCAGTAGC

D. simulans

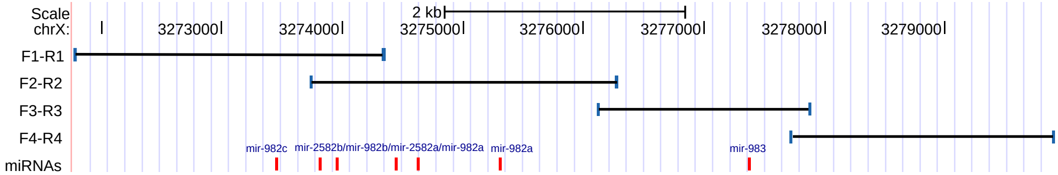

Primers

F1 AATGTGCCAATCCCAATGTT
F2 GATGGATTGATTTGATGATGGA
F3 GCCTGAATCTGGCCCGAAGT
F4 CTGCAGTACCTCTGCTCCAA

R1 ATTTACGCCCTTGAATGCAG
R2 TCATGGGACTAACCCGATTC
R3 GCTGAAATAGCGAATGAGCGTCT
R4 AGCAACAAACAGCTGCTCAA
