# Supplementary material for: New MicroRNAs in Drosophila—Birth, Death and Cycles of Adaptive Evolution
Source: PLoS Genet. 2014 Jan 23;10(1):e1004096. doi: 10.1371/journal.pgen.1004096 (PMC3900394; doi:10.1371/journal.pgen.1004096)
Supplement: Table S1 — The GEO accession numbers of the small RNA libraries used in this study. (PDF) [file pgen.1004096.s006.pdf]

Table S1. The GEO accession numbers of the small RNA libraries used in this study.

GEO accession	Species	Tissues/Development stages	Platform	Sources
GSM180328	*Drosophila melanogaster*	adult heads (female heads, male heads)	454	Ruby *et al*. 2007
GSM180329	*Drosophila melanogaster*	adult bodies (female bodies, male bodies)	454	Ruby *et al*. 2007
GSM180330	*Drosophila melanogaster*	very early embryo (0-1)	454	Ruby *et al*. 2007
GSM180331	*Drosophila melanogaster*	early embryo (2-6)	454	Ruby *et al*. 2007
GSM180332	*Drosophila melanogaster*	mid embryo (6-10)	454	Ruby *et al*. 2007
GSM180333	*Drosophila melanogaster*	late embryo (12-24)	454	Ruby *et al*. 2007
GSM180334	*Drosophila melanogaster*	larvae: 1st instar and 3rd instars	454	Ruby *et al*. 2007
GSM180335	*Drosophila melanogaster*	larval brain/gonads/imaginal discs	454	Ruby *et al*. 2007
GSM180336	*Drosophila melanogaster*	pupae: 0-1 day, 0-2 day, 2-4 day	454	Ruby *et al*. 2007
GSM180337	*Drosophila melanogaster*	tissue culture cells (S2 only)	454	Ruby *et al*. 2007
GSM246084	*Drosophila melanogaster*	adult male heads	454	Lu *et al*. 2008
GSM280082	*Drosophila melanogaster*	adult ovaries from 2-4 day old flies	solexa	Czech *et al*. 2008
GSM280085	*Drosophila melanogaster*	adult testes from 2-4 day old flies	solexa	Czech *et al*. 2008
GSM280088	*Drosophila melanogaster*	S2cell (AGO1IP)	solexa	Czech *et al*. 2008
GSM385744	*Drosophila melanogaster*	OSS cell replicates 1	solexa	Lau *et al*. 2009
GSM385748	*Drosophila melanogaster*	OSS cell replicates 2	solexa	Lau *et al*. 2009
GSM385821	*Drosophila melanogaster*	OSS cell replicates 3	solexa	Lau *et al*. 2009
GSM385822	*Drosophila melanogaster*	OSS cell replicates 4	solexa	Lau *et al*. 2009
GSM275691	*Drosophila melanogaster*	larval brain/gonads/imaginal discs	solexa	Chung *et al*. 2008
GSM286613	*Drosophila melanogaster*	0-1hr embryo replicates 2	solexa	Chung *et al*. 2008
GSM286604	*Drosophila melanogaster*	0-1hr embryo replicates 1	solexa	Chung *et al*. 2008
GSM286605	*Drosophila melanogaster*	2-6hr embryo replicates 1	solexa	Chung *et al*. 2008
GSM286606	*Drosophila melanogaster*	2-6hr embryo replicates 2	solexa	Chung *et al*. 2008
GSM286607	*Drosophila melanogaster*	6-10hr embryo replicates 1	solexa	Chung *et al*. 2008
GSM286611	*Drosophila melanogaster*	6-10hr embryo replicates 2	solexa	Chung *et al*. 2008
GSM286602	*Drosophila melanogaster*	adult male body	solexa	Chung *et al*. 2008
GSM286603	*Drosophila melanogaster*	adult female body	solexa	Chung *et al*. 2008
GSM240749	*Drosophila melanogaster*	adult female head	solexa	Chung *et al*. 2008
GSM286601	*Drosophila melanogaster*	adult male head	solexa	Chung *et al*. 2008
GSM364902	*Drosophila melanogaster*	12-24hr embryo	solexa	modENCODE
GSM322245	*Drosophila melanogaster*	3rd instar larvae replicates 2	solexa	modENCODE
GSM322208	*Drosophila melanogaster*	3rd instar larvae replicates 1	solexa	modENCODE
GSM322219	*Drosophila melanogaster*	2-4 day old pupae replicates 1	solexa	modENCODE
GSM322338	*Drosophila melanogaster*	2-4 day old pupae replicates 2	solexa	modENCODE
GSM399105	*Drosophila melanogaster*	larval brain/gonads/imaginal discs	solexa	modENCODE
GSM360256	*Drosophila melanogaster*	1st instar larvae replicates 1	solexa	modENCODE
GSM360257	*Drosophila melanogaster*	1st instar larvae replicates 2	solexa	modENCODE

GSM360260	*Drosophila melanogaster*	0-1 day old pupae	solexa	modENCODE
GSM360262	*Drosophila melanogaster*	0-2 day old pupae	solexa	modENCODE
GSM322533	*Drosophila melanogaster*	adult female head	solexa	modENCODE
GSM322543	*Drosophila melanogaster*	adult male head	solexa	modENCODE
GSM399106	*Drosophila melanogaster*	adult female body	solexa	modENCODE
GSM399107	*Drosophila melanogaster*	adult male body	solexa	modENCODE
GSM1165052	*Drosophila simulans*	adult ovaries from 3-5 day old flies	solexa	this study
GSM1165053	*Drosophila simulans*	adult testes from 3-5 day old flies	solexa	this study
GSM343915	*Drosophila simulans*	pooled 0-12 and 12-24 hour embryos	solexa	Berezikov *et al*. 2010
GSM246085	*Drosophila simulans*	adult male heads	454	Lu *et al*. 2008
GSM1165054	*Drosophila pseudoobscura*	adult ovaries from 3-5 day old flies	solexa	this study
GSM1165055	*Drosophila pseudoobscura*	adult testes from 3-5 day old flies	solexa	this study
GSM1165056	*Drosophila pseudoobscura*	larval brains and imaginal discs	solexa	this study
GSM343916	*Drosophila pseudoobscura*	pooled 0-12 and 12-24 hour embryos	solexa	Berezikov *et al*. 2010
GSM444067	*Drosophila pseudoobscura*	head	solexa	Berezikov *et al*. 2010
GSM246086	*Drosophila pseudoobscura*	adult male heads	454	Lu *et al*. 2008
GSM548599	*Drosophila virilis*	testes from Argentina strain	solexa	Rozhkov *et al*. 2010
GSM548600	*Drosophila virilis*	ovaries from Argentina strain	solexa	Rozhkov *et al*. 2010
GSM548603	*Drosophila virilis*	0-2hr embryos from 160 strain	solexa	Rozhkov *et al*. 2010
GSM548610	*Drosophila virilis*	testes from 9 strain	solexa	Rozhkov *et al*. 2010
GSM548613	*Drosophila virilis*	ovaries from 9 strain	solexa	Rozhkov *et al*. 2010
GSM548623	*Drosophila virilis*	testes from 160 strain	solexa	Rozhkov *et al*. 2010
GSM548627	*Drosophila virilis*	0-2hr embryos from 9 strain	solexa	Rozhkov *et al*. 2010
GSM548628	*Drosophila virilis*	ovaries from 160 strain	solexa	Rozhkov *et al*. 2010
