# Supplementary material for: New MicroRNAs in Drosophila—Birth, Death and Cycles of Adaptive Evolution
Source: PLoS Genet. 2014 Jan 23;10(1):e1004096. doi: 10.1371/journal.pgen.1004096 (PMC3900394; doi:10.1371/journal.pgen.1004096)
Supplement: Table S3 — miRNAs clustered in miR-982s, miR-310s, and miR-972s in D. melanogaster. (PDF) [file pgen.1004096.s008.pdf]

Table S3. miRNAs clustered in miR-982s, miR-310s, and miR-972s in *D. melanogaster*.

cluster	miRNA	age
miR-982s	dme-miR-984	0 - 4 Myrs
	dme-miR-983-2	0 - 4 Myrs
	dme-miR-983-1	4 - 30 Myrs
	dme-miR-303	4 - 30 Myrs
	dme-miR-982	4 - 30 Myrs
miR-310s	dme-miR-992	4 - 30 Myrs
	dme-miR-991	30 - 60 Myrs
	dme-miR-2498	4 - 30 Myrs
	dme-miR-313	60 - 250 Myrs
	dme-miR-312	30 - 60 Myrs
	dme-miR-311	60 - 250 Myrs
	dme-miR-310	60 - 250 Myrs
miR-972s	dme-miR-972	4 - 30 Myrs
	dme-miR-973	60 - 250 Myrs
	dme-miR-974	60 - 250 Myrs
	dme-miR-2499	4 - 30 Myrs
	dme-miR-4966	0 - 4 Myrs
	dme-miR-975	60 - 250 Myrs
	dme-miR-976	60 - 250 Myrs
	dme-miR-977	60 - 250 Myrs
	dme-miR-978	4 - 30 Myrs
	dme-miR-979	0 - 4 Myrs
