# Supplementary material for: New MicroRNAs in Drosophila—Birth, Death and Cycles of Adaptive Evolution
Source: PLoS Genet. 2014 Jan 23;10(1):e1004096. doi: 10.1371/journal.pgen.1004096 (PMC3900394; doi:10.1371/journal.pgen.1004096)
Supplement: Table S4 — Fly strains used for population sequencing of the miR-982s cluster. (PDF) [file pgen.1004096.s009.pdf]

Table S4. Fly strains used for population sequencing of the miR-982s cluster.

ID	Origin
D.melanogaster M line	
mm1	North Carolina, USA
mm17	North Carolina, USA
mm131	North Carolina, USA
mm140	North Carolina, USA
M-97	Miami, USA
M-103	Miami, USA
Floral city#13	Florida, USA
Oahu	Oahu, Hawaii
CanS, Canton, OH	Canton, Ohio, USA
Ica	Peru
EC158	Ecuador
EC175	Ecuador
FrV2-1	France
FrV3-1	France
HB99	Australia
FR8	Australia
Yv101	Australia
SFS2-11	Israel
SFS2-15	Israel
NFS6-3	Israel
NFS6-7	Israel
NFS6-10	Israel
Hg84(2)	Highgrove, California, USA
Hg84(3)	Highgrove, California, USA
India female weak Mysore	India
QD-12	Japan
QD-18	Japan
TWN-9	Taiwan
TWN-38	Taiwan
D.melanogaster Z line	
Zs2	Sangawa, Zimbabwe
Zs8	Sangawa, Zimbabwe
Zs11	Sangawa, Zimbabwe
Zs30(xz)	Sangawa, Zimbabwe
Zs53(xz)	Sangawa, Zimbabwe
Zs56	Sangawa, Zimbabwe
ZH12	Harare, Zimbabwe
ZH16	Harare, Zimbabwe
ZH18	Harare, Zimbabwe

ZH21	Harare, Zimbabwe
ZH34	Harare, Zimbabwe
mw28	Malawi, Africa
mw56	Malawi, Africa
D.simulans	
Davis, CA	Davis, CA
Morro Bay, CA	Morro Bay, CA
Floral City, FL	Floral City, FL
Latana, FL	Latana, FL
Hawaii	Hawaii
CookIS, Pacific Ocean	CookIS, Pacific Ocean
Reunion, Indian Ocean	Reunion, Indian Ocean
Arimiddle Woods, Aus	Australia
Canner Rive, Aus	Australia
Eden32, Aus	Australia
Kyogle4, Aus	Australia
Morya2, Aus	Australia
N.Caled48s	Australia
New Guinea	New Guinea
Lima	Lima, Peru
Tamiaguadala	Tamiaguadala, Mexico
Trinadad	Trinadad
W54	France
W60	Mishima Japan
W62	Ogasawana Japan
W89	Kenya
W81	Madagascar
Sim6	-
C167.4	-
MD106TS	-
