# Supplementary material for: New MicroRNAs in Drosophila—Birth, Death and Cycles of Adaptive Evolution
Source: PLoS Genet. 2014 Jan 23;10(1):e1004096. doi: 10.1371/journal.pgen.1004096 (PMC3900394; doi:10.1371/journal.pgen.1004096)
Supplement: Table S5 — Estimates of proportions of selection regimes on different classes of miRNA genes. Both canonical miRNAs and mirtrons from different age groups were classified into two subgroups according to their expression levels: low (≤200 RPM) and high (>200 PRM). The number of genes in each category is given in the parentheses. D, divergence between D. melanogaster and D. simulans; P, polymorphism in populations of D. melanogaster (DPGP); DAF, derived allele frequency; α, the fraction of adaptive fixations; b, the fraction of new mutants that are weakly deleterious; d, the fraction of new mutants that are strongly deleterious; f, the expected number of neutral segregating sites. (PDF) [file pgen.1004096.s010.pdf]

Table S5. Estimates of proportions of different classes of miRNA genes under positive selection. Both canonical miRNAs and mirtrons from different age groups were classified into two subgroups according to their expression levels: low (⩽ 200 RPM) and high (>200 PRM). The number of genes in each category is given in the parentheses. D, divergence between *D. melanogaster* and *D. simulans*; P, polymorphism in populations of *D. melanogaster* (DPGP); DAF, derived allele frequency; α, the fraction of adaptive fixations; b, the fraction of new mutants that are weakly deleterious; d, the fraction of new mutants that are strongly deleterious; f, the expected number of neutral segregating sites.

Site type		Site number	D	P (DAF>5%)	P (DAF<5%)	α (%)	b (%)	d (%)	f (%)
4-fold degenerate sites		3,495,672	378,361	83,996	84,983				
canonical miRNAs									
0-4 Myrs	low (60)	5,692	271	48	137	-15.3	15.4	33.9	50.7
	high (12)	1,118	86	4	4	79.1	0.0	6.1	14.8
4-30 Myrs	low (2)	192	8	1	3	(n. a.)	(n. a.)	(n. a.)	(n. a.)
	high (10)	944	110	3	6	84.7	3.2	-4.4	16.5
30-60 Myrs	low (3)	287	3	1	0	(n. a.)	(n. a.)	(n. a.)	(n. a.)
	high (9)	800	35	12	7	-12.2	-3.6	82.2	10.8
60-250 Myrs	low (3)	190	4	3	4	(n. a.)	(n. a.)	(n. a.)	(n. a.)
	high (34)	2,931	114	14	23	36.0	3.1	37.9	23.0
>250 Myrs	low (0)	0	0	0	0	(n. a.)	(n. a.)	(n. a.)	(n. a.)
	high (71)	6,355	72	18	42	-49.8	3.8	80.5	15.7
mirtrons									
0-4 Myrs	low (21)	1,372	105	13	24	32.6	8.1	11.6	47.7
	high (3)	355	11	9	5	-199.3	-21.2	-4.5	125.7
4-30 Myrs	low (3)	176	2	1	6	(n. a.)	(n. a.)	(n. a.)	(n. a.)
	high (2)	116	2	1	1	(n. a.)	(n. a.)	(n. a.)	(n. a.)
30-60 Myrs	low (1)	71	2	0	1	(n. a.)	(n. a.)	(n. a.)	(n. a.)
	high (1)	68	0	2	1	(n. a.)	(n. a.)	(n. a.)	(n. a.)
60-250 Myrs	low (0)	0	0	0	0	(n. a.)	(n. a.)	(n. a.)	(n. a.)
	high (3)	191	0	3	4	(n. a.)	(n. a.)	(n. a.)	(n. a.)
>250 Myrs	low (0)	0	0	0	0	(n. a.)	(n. a.)	(n. a.)	(n. a.)
	high (0)	0	0	0	0	(n. a.)	(n. a.)	(n. a.)	(n. a.)
