# Supplementary material for: New MicroRNAs in Drosophila—Birth, Death and Cycles of Adaptive Evolution
Source: PLoS Genet. 2014 Jan 23;10(1):e1004096. doi: 10.1371/journal.pgen.1004096 (PMC3900394; doi:10.1371/journal.pgen.1004096)
Supplement: Table S6 — Fst of each miRNA from miR-982s between M-line and Z-line. (PDF) [file pgen.1004096.s011.pdf]

Table S6. Fst of each miRNA from miR-982s between M-line and Z-line

miRNA	Fst
dme-miR-984	0.318
dme-miR-983-2	0.0615
dme-miR-983-1	0.0189
dme-miR-303	0.252
dme-miR-982	0.000
