# Supplementary material for: New MicroRNAs in Drosophila—Birth, Death and Cycles of Adaptive Evolution
Source: PLoS Genet. 2014 Jan 23;10(1):e1004096. doi: 10.1371/journal.pgen.1004096 (PMC3900394; doi:10.1371/journal.pgen.1004096)
Supplement: Table S7 — KmiR/KS of the older miRNAs (60–250 Myrs) that have been evolving rapidly between D. melanogaster and D. simulans. The common ancestral sequences of D. melanogaster and D. simulans are inferred from D. yakuba and D. erecta. (PDF) [file pgen.1004096.s012.pdf]

Table S7. K_{miR} / Ks of the older miRNAs (60-250 Myrs) that have been evolving rapidly between *D. melanogaster* and *D. simulans*. The common ancestral sequences of *D. melanogaster* and *D. simulans* are inferred from *D. yakuba* and *D. erecta*.

miRNA	KmiR / Ks (*D. mel* vs the common ancestor of *D. mel* and D. sim)	KmiR / Ks (*D. sim* vs the common ancestor of *D. mel* and *D. sim*)
dme-mir-311	0.814	0.194
dme-mir-313	0.475	1.231
dme-mir-964	0.680	0.140
dme-mir-973	0.511	1.676
dme-mir-974	0.676	0.862
dme-mir-975	0.184	0.943
dme-mir-976	0.346	0.743
dme-mir-977	0.687	1.290
