# Supplementary material for: New MicroRNAs in Drosophila—Birth, Death and Cycles of Adaptive Evolution
Source: PLoS Genet. 2014 Jan 23;10(1):e1004096. doi: 10.1371/journal.pgen.1004096 (PMC3900394; doi:10.1371/journal.pgen.1004096)
Supplement: Table S8 — Age and evolutionary mode of the 136 highly expressed, canonical miRNAs analyzed in this study. (PDF) [file pgen.1004096.s013.pdf]

Table S8. Age and evolutionary mode of the 136 highly expressed, canonical miRNAs analyzed in this study.

miRNA	Age	Evolutionary mode
dme-mir-1017	0 - 4 Myrs	adaptive
dme-mir-2279	0 - 4 Myrs	adaptive
dme-mir-4966	0 - 4 Myrs	adaptive
dme-mir-954	0 - 4 Myrs	adaptive
dme-mir-956	0 - 4 Myrs	adaptive
dme-mir-971	0 - 4 Myrs	adaptive
dme-mir-979	0 - 4 Myrs	adaptive
dme-mir-983-2	0 - 4 Myrs	adaptive
dme-mir-984	0 - 4 Myrs	adaptive
dme-mir-985	0 - 4 Myrs	adaptive
dme-mir-990	0 - 4 Myrs	adaptive
dme-mir-997	0 - 4 Myrs	adaptive
dme-mir-1001	4 - 30 Myrs	adaptive
dme-mir-2494	4 - 30 Myrs	adaptive
dme-mir-2498	4 - 30 Myrs	adaptive
dme-mir-2499	4 - 30 Myrs	adaptive
dme-mir-303	4 - 30 Myrs	adaptive
dme-mir-972	4 - 30 Myrs	adaptive
dme-mir-978	4 - 30 Myrs	adaptive
dme-mir-982	4 - 30 Myrs	adaptive
dme-mir-983-1	4 - 30 Myrs	adaptive
dme-mir-992	4 - 30 Myrs	adaptive
dme-mir-1002	30 - 60 Myrs	conservative
dme-mir-2b-1	30 - 60 Myrs	conservative
dme-mir-312	30 - 60 Myrs	transitional
dme-mir-959	30 - 60 Myrs	conservative
dme-mir-960	30 - 60 Myrs	conservative
dme-mir-961	30 - 60 Myrs	transitional
dme-mir-968	30 - 60 Myrs	conservative
dme-mir-986	30 - 60 Myrs	conservative
dme-mir-991	30 - 60 Myrs	transitional
dme-mir-13b-2	60 - 250 Myrs	conservative
dme-mir-274	60 - 250 Myrs	conservative
dme-mir-281-2	60 - 250 Myrs	conservative
dme-mir-284	60 - 250 Myrs	conservative
dme-mir-2a-2	60 - 250 Myrs	conservative
dme-mir-304	60 - 250 Myrs	conservative
dme-mir-309	60 - 250 Myrs	conservative

dme-mir-310	60 - 250 Myrs	conservative
dme-mir-311	60 - 250 Myrs	transitional
dme-mir-313	60 - 250 Myrs	transitional
dme-mir-314	60 - 250 Myrs	conservative
dme-mir-318	60 - 250 Myrs	conservative
dme-mir-31b	60 - 250 Myrs	conservative
dme-mir-33	60 - 250 Myrs	conservative
dme-mir-4	60 - 250 Myrs	conservative
dme-mir-5	60 - 250 Myrs	conservative
dme-mir-6-1	60 - 250 Myrs	conservative
dme-mir-6-2	60 - 250 Myrs	conservative
dme-mir-6-3	60 - 250 Myrs	conservative
dme-mir-955	60 - 250 Myrs	conservative
dme-mir-958	60 - 250 Myrs	conservative
dme-mir-962	60 - 250 Myrs	conservative
dme-mir-963	60 - 250 Myrs	conservative
dme-mir-964	60 - 250 Myrs	transitional
dme-mir-969	60 - 250 Myrs	conservative
dme-mir-973	60 - 250 Myrs	adaptive
dme-mir-974	60 - 250 Myrs	adaptive
dme-mir-975	60 - 250 Myrs	adaptive
dme-mir-976	60 - 250 Myrs	adaptive
dme-mir-977	60 - 250 Myrs	transitional
dme-mir-980	60 - 250 Myrs	conservative
dme-mir-987	60 - 250 Myrs	conservative
dme-mir-994	60 - 250 Myrs	conservative
dme-mir-995	60 - 250 Myrs	conservative
dme-bantam	> 250 Myrs	conservative
dme-let-7	> 250 Myrs	conservative
dme-mir-1	> 250 Myrs	conservative
dme-mir-10	> 250 Myrs	conservative
dme-mir-100	> 250 Myrs	conservative
dme-mir-1000	> 250 Myrs	conservative
dme-mir-11	> 250 Myrs	conservative
dme-mir-12	> 250 Myrs	conservative
dme-mir-124	> 250 Myrs	conservative
dme-mir-125	> 250 Myrs	conservative
dme-mir-133	> 250 Myrs	conservative
dme-mir-137	> 250 Myrs	conservative
dme-mir-13a	> 250 Myrs	conservative
dme-mir-13b-1	> 250 Myrs	conservative
dme-mir-14	> 250 Myrs	conservative
dme-mir-184	> 250 Myrs	conservative

dme-mir-190	> 250 Myrs	conservative
dme-mir-193	> 250 Myrs	conservative
dme-mir-210	> 250 Myrs	conservative
dme-mir-219	> 250 Myrs	conservative
dme-mir-252	> 250 Myrs	conservative
dme-mir-263a	> 250 Myrs	conservative
dme-mir-263b	> 250 Myrs	conservative
dme-mir-275	> 250 Myrs	conservative
dme-mir-276a	> 250 Myrs	conservative
dme-mir-276b	> 250 Myrs	conservative
dme-mir-277	> 250 Myrs	conservative
dme-mir-278	> 250 Myrs	conservative
dme-mir-279	> 250 Myrs	conservative
dme-mir-281-1	> 250 Myrs	conservative
dme-mir-282	> 250 Myrs	conservative
dme-mir-283	> 250 Myrs	conservative
dme-mir-285	> 250 Myrs	conservative
dme-mir-286	> 250 Myrs	conservative
dme-mir-2a-1	> 250 Myrs	conservative
dme-mir-2b-2	> 250 Myrs	conservative
dme-mir-2c	> 250 Myrs	conservative
dme-mir-3	> 250 Myrs	conservative
dme-mir-305	> 250 Myrs	conservative
dme-mir-306	> 250 Myrs	conservative
dme-mir-307a	> 250 Myrs	conservative
dme-mir-308	> 250 Myrs	conservative
dme-mir-315	> 250 Myrs	conservative
dme-mir-316	> 250 Myrs	conservative
dme-mir-317	> 250 Myrs	conservative
dme-mir-31a	> 250 Myrs	conservative
dme-mir-34	> 250 Myrs	conservative
dme-mir-375	> 250 Myrs	conservative
dme-mir-7	> 250 Myrs	conservative
dme-mir-79	> 250 Myrs	conservative
dme-mir-8	> 250 Myrs	conservative
dme-mir-87	> 250 Myrs	conservative
dme-mir-927	> 250 Myrs	conservative
dme-mir-929	> 250 Myrs	conservative
dme-mir-92a	> 250 Myrs	conservative
dme-mir-92b	> 250 Myrs	conservative
dme-mir-932	> 250 Myrs	conservative
dme-mir-957	> 250 Myrs	conservative
dme-mir-965	> 250 Myrs	conservative

dme-mir-970	> 250 Myrs	conservative
dme-mir-981	> 250 Myrs	conservative
dme-mir-988	> 250 Myrs	conservative
dme-mir-989	> 250 Myrs	conservative
dme-mir-993	> 250 Myrs	conservative
dme-mir-996	> 250 Myrs	conservative
dme-mir-998	> 250 Myrs	conservative
dme-mir-999	> 250 Myrs	conservative
dme-mir-9a	> 250 Myrs	conservative
dme-mir-9b	> 250 Myrs	conservative
dme-mir-9c	> 250 Myrs	conservative
dme-mir-iab-4	> 250 Myrs	conservative
