# Supplementary material for: New MicroRNAs in Drosophila—Birth, Death and Cycles of Adaptive Evolution
Source: PLoS Genet. 2014 Jan 23;10(1):e1004096. doi: 10.1371/journal.pgen.1004096 (PMC3900394; doi:10.1371/journal.pgen.1004096)
Supplement: Table S9 — GO enrichment of the predicted target genes of miR-982s in D. melanogaster and D. simulans. Targets were predicted by seed match using TargetScan (v5.0 http://www.targetscan.org/fly_12/) [5]. (PDF) [file pgen.1004096.s014.pdf]

Table S9. GO enrichment of predicted target genes of miR-982s in *D. melanogaster* and *D. simulans*. Targets were predicted by seed match using TargetScan (v5.0 http://www.targetscan.org/fly_12/)

D. melanogaster

Term	P-value	Fold Enrichment
GO:0048580~regulation of post-embryonic development	0.025907537	11.12663551
GO:0040034~regulation of development, heterochronic	0.018457496	6.743415463
GO:0007584~response to nutrient	0.005369981	6.62299733
GO:0048065~male courtship behavior, veined wing extension	0.02953808	5.70596693
GO:0045197~establishment or maintenance of epithelial cell apical/basal polarity	0.02013444	4.636098131
GO:0009991~response to extracellular stimulus	0.009563521	4.450654206
GO:0031667~response to nutrient levels	0.009563521	4.450654206
GO:0007030~Golgi organization	0.023843144	4.415331553
GO:0016203~muscle attachment	0.037313864	3.863415109
GO:0007530~sex determination	0.042599643	3.708878505
GO:0035088~establishment or maintenance of apical/basal cell polarity	0.048285081	3.566229331
GO:0006576~biogenic amine metabolic process	0.037717021	3.179038718
GO:0009309~amine biosynthetic process	0.038820176	2.761930801
GO:0045664~regulation of neuron differentiation	0.02468091	2.747317411
GO:0050767~regulation of neurogenesis	0.02649637	2.491037802
GO:0016311~dephosphorylation	0.013843041	2.211716539
GO:0051056~regulation of small GTPase mediated signal transduction	0.029073381	2.170088487
GO:0042048~olfactory behavior	0.031001251	2.14724545
GO:0007422~peripheral nervous system development	0.046243933	2.107317332
GO:0007635~chemosensory behavior	0.03395266	2.023024639

D. simulans

Term	P-value	Fold Enrichment
GO:0042387~plasmatocyte differentiation	0.027146793	4.998110831
GO:0035209~pupal development	0.027146793	4.998110831
GO:0007432~salivary gland boundary specification	9.79E-07	4.331696054
GO:0007451~dorsal/ventral lineage restriction, imaginal disc	0.016905841	4.165092359
GO:0046667~compound eye retinal cell programmed cell death	0.016905841	4.165092359
GO:0006898~receptor-mediated endocytosis	8.56E-04	3.998488665
GO:0070304~positive regulation of stress-activated protein kinase signaling pathway	0.003044561	3.887419535
GO:0043410~positive regulation of MAPKKK cascade	0.003044561	3.887419535
GO:0046330~positive regulation of JNK cascade	0.003044561	3.887419535
GO:0010740~positive regulation of protein kinase cascade	0.001947101	3.634989695

GO:0042133~neurotransmitter metabolic process	0.033250101	3.570079165
GO:0035073~pupariation	0.033250101	3.570079165
GO:0043112~receptor metabolic process	0.033250101	3.570079165
GO:0042688~crystal cell differentiation	0.033250101	3.570079165
GO:0035111~leg joint morphogenesis	0.003869515	3.332073887
GO:0022407~regulation of cell-cell adhesion	0.019498262	3.332073887
GO:0007041~lysosomal transport	0.002370883	3.213071249
GO:0040034~regulation of development, heterochronic	0.011584705	3.180615984
GO:0007034~vacuolar transport	8.96E-04	3.054401064
GO:0048645~organ formation	1.85E-07	2.998866499
GO:0035078~induction of programmed cell death by ecdysone	0.032668694	2.998866499
GO:0035161~imaginal disc lineage restriction	0.032668694	2.998866499
GO:0048859~formation of anatomical boundary	7.47E-08	2.953429128
GO:0007416~synaptogenesis	3.41E-04	2.953429128
GO:0035285~appendage segmentation	0.00254843	2.940065195
GO:0035286~leg segmentation	0.00254843	2.940065195
GO:0010160~formation of organ boundary	1.56E-06	2.893643113
GO:0007370~ventral furrow formation	0.011524722	2.856063332
GO:0007479~leg disc proximal/distal pattern formation	0.006936849	2.811437343
GO:0035223~leg disc pattern formation	0.006936849	2.811437343
GO:0050770~regulation of axonogenesis	0.004201044	2.77672824
GO:0042386~hemocyte differentiation	0.004201044	2.77672824
GO:0007427~epithelial cell migration, open tracheal system	1.37E-04	2.75757839
GO:0010631~epithelial cell migration	1.37E-04	2.75757839
GO:0045610~regulation of hemocyte differentiation	0.001561972	2.726242272
GO:0001710~mesodermal cell fate commitment	0.017937059	2.66565911
GO:0048333~mesodermal cell differentiation	0.017937059	2.66565911
GO:0001667~ameboidal cell migration	1.38E-04	2.655246379
GO:0048584~positive regulation of response to stimulus	0.010837665	2.646058675
GO:0035050~embryonic heart tube development	0.010837665	2.646058675
GO:0007016~cytoskeletal anchoring at plasma membrane	0.010837665	2.646058675
GO:0048741~skeletal muscle fiber development	0.002458278	2.607709999
GO:0007528~neuromuscular junction development	0.002458278	2.607709999
GO:0035110~leg morphogenesis	5.23E-05	2.56313376
GO:0050808~synapse organization	5.05E-05	2.499055416
GO:0001763~morphogenesis of a branching structure	5.05E-05	2.499055416
GO:0035108~limb morphogenesis	8.11E-05	2.499055416
GO:0060173~limb development	8.11E-05	2.499055416
GO:0031344~regulation of cell projection organization	1.30E-04	2.499055416
GO:0010975~regulation of neuron projection development	8.78E-04	2.499055416
GO:0010769~regulation of cell morphogenesis involved in	8.78E-04	2.499055416

differentiation		
GO:0008345~larval locomotory behavior	0.026492778	2.499055416
GO:0035146~tube fusion	0.026492778	2.499055416
GO:0035147~branch fusion, open tracheal system	0.026492778	2.499055416
GO:0030239~myofibril assembly	0.043659414	2.499055416
GO:0032012~regulation of ARF protein signal transduction	0.043659414	2.499055416
GO:0055001~muscle cell development	4.79E-05	2.443520851
GO:0055002~striated muscle cell development	4.79E-05	2.443520851
GO:0048754~branching morphogenesis of a tube	1.97E-04	2.434977072
GO:0060446~branching involved in open tracheal system development	1.97E-04	2.434977072
GO:0048747~muscle fiber development	0.00131031	2.418440725
GO:0007480~imaginal disc-derived leg morphogenesis	0.002107887	2.412881091
GO:0035127~post-embryonic limb morphogenesis	0.002107887	2.412881091
GO:0048332~mesoderm morphogenesis	0.003394287	2.406497808
GO:0007469~antennal development	0.008831534	2.390400832
GO:0060562~epithelial tube morphogenesis	1.83E-04	2.380052777
GO:0007449~proximal/distal pattern formation, imaginal disc	0.014273482	2.380052777
GO:0035162~embryonic hemopoiesis	0.023102581	2.367526183
GO:0035218~leg disc development	4.17E-05	2.352052156
GO:0007303~cytoplasmic transport, nurse cell to oocyte	0.037451356	2.352052156
GO:0035109~imaginal disc-derived limb morphogenesis	0.003048077	2.332451721
GO:0001707~mesoderm formation	0.007798783	2.306820384
GO:0016203~muscle attachment	0.01247673	2.290800798
GO:0007478~leg disc morphogenesis	0.004303132	2.257211343
GO:0008593~regulation of Notch signaling pathway	0.01714671	2.199168766
GO:0060537~muscle tissue development	0.001336273	2.19429256
GO:0016055~Wnt receptor signaling pathway	1.46E-05	2.182273743
GO:0048545~response to steroid hormone stimulus	0.027112957	2.173091666
GO:0009954~proximal/distal pattern formation	0.027112957	2.173091666
GO:0035075~response to ecdysone	0.027112957	2.173091666
GO:0008406~gonad development	0.009348848	2.165848027
GO:0048608~reproductive structure development	0.009348848	2.165848027
GO:0032318~regulation of Ras GTPase activity	0.005150012	2.142047499
GO:0007519~skeletal muscle tissue development	0.005150012	2.142047499
GO:0007494~midgut development	0.014677979	2.142047499
GO:0016339~calcium-dependent cell-cell adhesion	0.042778473	2.142047499
GO:0048568~embryonic organ development	0.042778473	2.142047499
GO:0045664~regulation of neuron differentiation	3.71E-04	2.128824984
GO:0014706~striated muscle tissue development	0.00285835	2.124197103
GO:0032483~regulation of Rab protein signal transduction	0.022989164	2.114585352
GO:0032313~regulation of Rab GTPase activity	0.022989164	2.114585352

GO:0060538~skeletal muscle organ development	3.25E-04	2.104467718
GO:0048190~wing disc dorsal/ventral pattern formation	8.94E-04	2.099206549
GO:0050767~regulation of neurogenesis	1.04E-04	2.088762735
GO:0030716~oocyte fate determination	0.035894693	2.08254618
GO:0048542~lymph gland development	0.035894693	2.08254618
GO:0035295~tube development	2.27E-06	2.069924688
GO:0030952~establishment or maintenance of cytoskeleton polarity	0.019474859	2.068183792
GO:0030951~establishment or maintenance of microtubule cytoskeleton polarity	0.019474859	2.068183792
GO:0016325~oocyte microtubule cytoskeleton organization	0.019474859	2.068183792
GO:0048565~gut development	7.93E-05	2.05401815
GO:0048619~embryonic hindgut morphogenesis	0.016491083	2.030482525
GO:0035239~tube morphogenesis	3.41E-05	2.02304486
GO:0015837~amine transport	0.005062976	2.02304486
GO:0001708~cell fate specification	5.05E-04	2.015367271
GO:0007369~gastrulation	1.62E-04	2.013127974
