# Supplementary material for: New MicroRNAs in Drosophila—Birth, Death and Cycles of Adaptive Evolution
Source: PLoS Genet. 2014 Jan 23;10(1):e1004096. doi: 10.1371/journal.pgen.1004096 (PMC3900394; doi:10.1371/journal.pgen.1004096)
Supplement: Table S10 — PCR primers of the miR-982s cluster. (PDF) [file pgen.1004096.s015.pdf]

Table S10. PCR primers of the miR-982s cluster.

Primer	Sequence (5'-3')
cDNA PCR primers for *D.yakuba*	
dya-miR-2582-anc-F	TTCTTGGAAGCAGTCCTAAA
dya-miR-2582-anc-R	GAACAAAATGACAGTGCCTT
dya-miR-303-anc-F	ACGATGCGAAATAATGCTGA
dya-miR-303-anc-R	ACAACCACATGGGACCAAA
cDNA PCR primers for *D. erecta*	
der-miR-982-anc-F	TTCGCAAGAAACCAAATG
der-miR-982-anc-R	AATGAAATGGTTTTAGTGCTG
der-miR-983-anc-F	CTACCCGCAACTCAAGGA
der-miR-983-anc-R	TCGATTCGAAGTTATGTGCT
Population sequencing primers for *D. melanogaster*	
dme-miR-982s-F1	TAACCAACGCCTACGACTAT
dme-miR-982s-R1	TCTAACATTTGGGCTCATTT
dme-miR-982s-F2	AGCCTCATATCACCGCTGTC
dme-miR-982s-R2	GAAACTGCATTCCCCGAACG
dme-miR-982s-F3	CAATGGCGTAAATGGTTGTG
dme-miR-982s-R3	CGTGTGCAAATAGTGGTGCT
dme-miR-982s-F4	TGCCATCAAGAAGATTCACAGCG
dme-miR-982s-R4	CGCTGTGAATCTTCTTGATGGCA
dme-miR-982s-F5	AAGCCATTCCCATTCACTCAC
dme-miR-982s-R5	AGACACCGACCACCAGTAGC
Population sequencing primers for *D. simulans*	
dsi-miR-982s-F1	AATGTGCCAATCCCAATGTT
dsi-miR-982s-R1	ATTTACGCCCTTGAATGCAG
dsi-miR-982s-F2	GATGGATTGATTTGATGATGGA
dsi-miR-982s-R2	TCATGGGACTAACCCGATTC
dsi-miR-982s-F3	GCCTGAATCTGGCCCGAAGT
dsi-miR-982s-R3	GCTGAAATAGCGAATGAGCGTCT
dsi-miR-982s-F4	CTGCAGTACCTCTGCTCCAA
dsi-miR-982s-R4	AGCAACAAACAGCTGCTCAA
