# Supplementary material for: New MicroRNAs in Drosophila—Birth, Death and Cycles of Adaptive Evolution
Source: PLoS Genet. 2014 Jan 23;10(1):e1004096. doi: 10.1371/journal.pgen.1004096 (PMC3900394; doi:10.1371/journal.pgen.1004096)
Supplement: Text S1 — Interpretation of McDonald-Kreitman test result. (PDF) [file pgen.1004096.s016.pdf]

Supporting Text

Text S1. Interpretation of McDonald-Kreitman test result.

In the standard interpretation of the MK test [1], a higher D/P ratio of A than that of S would be attributed to an increase in divergence, rather than a decrease in polymorphism. It would then suggest positive selection. However, as Fay, Wyckoff and Wu [2] pointed out, an increase in selective constraint (e.g. due to a recent increase in the effective population size) could also lead an increase in D/P, but through a reduction in polymorphism instead of an increase in divergence. This would seem plausible in *Drosophila melanogaster* which may have undergone a recent population expansion [3].

To test this alternative explanation, we checked whether the increase in D/P among new miRNAs was due to an increase in divergence or a decrease in polymorphism. We found new miRNAs (<30 Myrs) and old miRNAs (> 30 Myrs) are comparably polymorphic; hence, the increase in D/P must be due to an excess of divergence in these new miRNAs (figure below). Positive selection driving miRNA divergence is a more likely explanation.

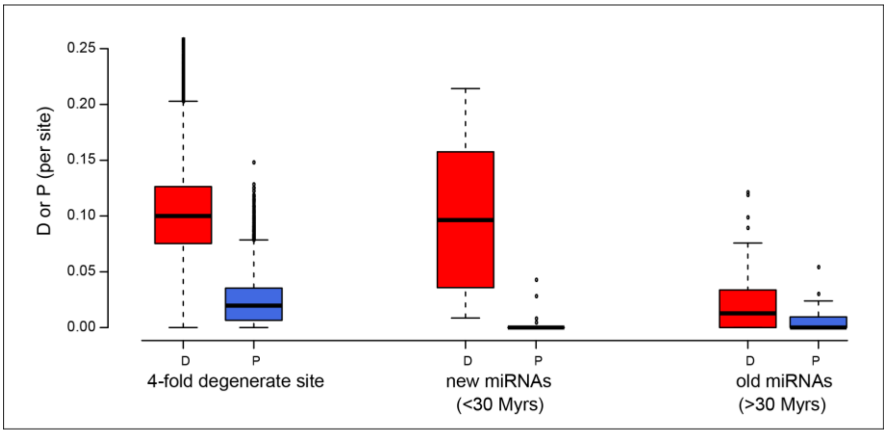

References

1. McDonald JH, Kreitman M (1991) Adaptive protein evolution at the Adh locus in *Drosophila*. Nature 351: 652-654.

2. Fay JC, Wyckoff GJ, Wu CI (2002) Testing the neutral theory of molecular evolution with genomic data from *Drosophila*. Nature 415: 1024-1026.

3. Stephan W, Li H (2007) The recent demographic and adaptive history of *Drosophila melanogaster*. Heredity 98: 65-68.
