# Supplementary material for: New MicroRNAs in Drosophila—Birth, Death and Cycles of Adaptive Evolution
Source: PLoS Genet. 2014 Jan 23;10(1):e1004096. doi: 10.1371/journal.pgen.1004096 (PMC3900394; doi:10.1371/journal.pgen.1004096)
Supplement: Text S2 — An alternative explanation for expression evolution of new and old miRNAs. (PDF) [file pgen.1004096.s017.pdf]

Supporting Text

Text S2. An alternative explanation for expression evolution of new and old miRNAs.

An alternative explanation is that lowly and highly expressed miRNAs have different birth and death rates. If highly expressed miRNAs have a lower birth and death rate than lowly expressed miRNAs, we would expect more highly expressed miRNAs in the old groups. This alternative explanation is strongly suggested by an anonymous reviewer. However, it is difficult for a new miRNA to acquire a high and broad expression immediately after birth. If it does, it is difficult for it to avoid being strongly deleterious [1,2]. At this moment, we cannot definitively affirm a single hypothesis for the contrast between old and new miRNAs.

References

1. Tang T, Kumar S, Shen Y, Lu J, Wu ML, et al. (2010) Adverse interactions between micro-RNAs and target genes from different species. Proc Natl Acad Sci U S A 107: 12935-12940.

2. Shen Y, Lv Y, Huang L, Liu W, Wen M, et al. (2011) Testing hypotheses on the rate of molecular evolution in relation to gene expression using microRNAs. Proc Natl Acad Sci U S A 108: 15942-15947.
