# Supplementary material for: New MicroRNAs in Drosophila—Birth, Death and Cycles of Adaptive Evolution
Source: PLoS Genet. 2014 Jan 23;10(1):e1004096. doi: 10.1371/journal.pgen.1004096 (PMC3900394; doi:10.1371/journal.pgen.1004096)
Supplement: Text S3 — The evolution of miR-982s, miR-310s and miR-972s expression. (PDF) [file pgen.1004096.s018.pdf]

Supporting Text

Text S3. The evolution of miR-982s, miR-310s and miR-972s expression.

The young miR-982s (dme-miR-982/303/983-1/983-2/984) are expressed in testes at high or moderate levels. In the mixed-age miR-310s and miR-972s, young members (dme-miR-991/992/2498 from miR-310s and dme-miR-979/4966 from miR-972s, see also **Table S3**) are preferentially expressed in testes and larval brain/gonads/imaginal discs, whereas old members (dme-miR-310/311/312/313 from miR-310s and dme-miR-973/974/975/976/977 from miR-972s, see also **Table S2**) have broadened their expression to other tissues such as embryos, ovaries and the central neuron system (CNS). In both clusters, the younger members have much lower breadth and magnitude of expression than the older members.
